# Identification of Quantitative Trait Loci Associated with Nutrient Use Efficiency Traits, Using SNP Markers in an Early Backcross Population of Rice (*Oryza sativa* L.)

**DOI:** 10.3390/ijms20040900

**Published:** 2019-02-19

**Authors:** Zilhas Ahmed Jewel, Jauhar Ali, Anumalla Mahender, Jose Hernandez, Yunlong Pang, Zhikang Li

**Affiliations:** 1 Rice Breeding Platform, International Rice Research Institute (IRRI), Los Baños, Laguna 4031, Philippines; jeweluplb@gmail.com (Z.A.J.); m.anumalla@irri.org (A.M.); y.pang@sdau.edu.cn (Y.P.); 2 College of Agriculture, University of the Philippines Los Baños, Laguna 4031, Philippines; joehernandez56@gmail.com; 3 College of Agronomy, Shandong Agricultural University, Taian 271018, China; 4 Institute of Crop Sciences, Chinese Academy of Agricultural Sciences, Beijing 100081, China; zhkli1953@126.com

**Keywords:** nutrient use efficiency, quantitative trait loci (QTLs), molecular markers, agronomic efficiency, partial factor productivity

## Abstract

The development of rice cultivars with nutrient use efficiency (NuUE) is highly crucial for sustaining global rice production in Asia and Africa. However, this requires a better understanding of the genetics of NuUE-related traits and their relationship to grain yield. In this study, simultaneous efforts were made to develop nutrient use efficient rice cultivars and to map quantitative trait loci (QTLs) governing NuUE-related traits in rice. A total of 230 BC_1_F_5_ introgression lines (ILs) were developed from a single early backcross population involving Weed Tolerant Rice 1, as the recipient parent, and Hao-an-nong, as the donor parent. The ILs were cultivated in field conditions with a different combination of fertilizer schedule under six nutrient conditions: minus nitrogen (–N), minus phosphorus (–P), (–NP), minus nitrogen phosphorus and potassium (–NPK), 75% of recommended nitrogen (75N), and NPK. Analysis of variance revealed that significant differences (*p* < 0.01) were noted among ILs and treatments for all traits. A high-density linkage map was constructed by using 704 high-quality single nucleotide polymorphism (SNP) markers. A total of 49 main-effect QTLs were identified on all chromosomes, except on chromosome 7, 11 and 12, which are showing 20.25% to 34.68% of phenotypic variation. With further analysis of these QTLs, we refined them to four top hotspot QTLs (QTL harbor-I to IV) located on chromosomes 3, 5, 9, and 11. However, we identified four novel putative QTLs for agronomic efficiency (AE) and 22 QTLs for partial factor productivity (PFP) under –P and 75N conditions. These interval regions of QTLs, several transporters and genes are located that were involved in nutrient uptake from soil to plant organs and tolerance to biotic and abiotic stresses. Further, the validation of these potential QTLs, genes may provide remarkable value for marker-aided selection and pyramiding of multiple QTLs, which would provide supporting evidence for the enhancement of grain yield and cloning of NuUE tolerance-responsive genes in rice.

## 1. Introduction

Rice (*Oryza sativa* L.) is one of the most prominent staple food crops in the world and it has significantly contributed to global food security [1]. By 2050, rice production has to be increased by more than 60% to meet the rapid increase in food demand [2,3]. Approximately 90% of rice cultivation is carried out in irrigated and lowland rainfed ecosystems in Asia, where we still face problems such as decreasing arable land and freshwater availability, and increased labor costs and biotic and abiotic stress factors that impede production and productivity. Further, the drastic rise in fertilizer costs drives the search for suitable rice cultivars that are efficient in grain yield production [4,5,6,7,8]. Nutrient fertilizers are one of the most predominant factors that influence the genetic enhancement of grain yield productivity in most agricultural regions [6].

In the last four decades, the amount of nitrogen (N) applied to crops has risen 12 to 104 teragrams per year (Tg year^−1^), and 30%–50% of the N has been harvested in the grain and the remaining 70%–50% of the fertilizer has been lost through a combination of leaching, denitrification, volatilization, surface runoff, and microbial consumption [9]. Therefore, the excessive amount and long-term use of fertilizers cause severe environmental pollution, and this is one of the significant costly inputs for poor farmers [10,11,12,13,14]. Hence, the identification of crops with higher NuUE that are less dependent on NPK fertilizers (nitrogen, phosphorous, and potassium) is crucial for the sustainability of agriculture. Kant et al. [15] estimated that an increase of 1% in NuUE could save about US$ 1.1 billion annually. The Green Revolution period witnessed a modest increase of 13% in harvested rice area in Asia, whereas grain yield production more than doubled, from 240 to 513 million tons, with consumption of fertilizers high in NPK increasing from 6.7 to 69.0 million tons. According to the FAO electronic database, particularly in Asian countries, China, Sri Lanka, Indonesia, and India had the highest average fertilizer consumption of 565.2 kg ha^−1^, 251.7 kg ha^−1^, 185.141 kg ha^−1^, and 157.48 kg ha^−1^, respectively, from 2005 to 2014 (http://ricestat.irri.org:8080/wrsv3/entrypoint.htm#). Therefore, the identification of superior cultivars with improved nutrient use efficiency (NuUE) is an essential target research area for plant breeders, for attaining higher grain yield and also reducing production costs [16].

In the improvement of the tolerance of rice cultivars of single biotic and abiotic stresses, tremendous progress has been achieved [8,17,18,19,20,21]. However, these tolerant varieties have not been able to succeed to attain higher grain yields under both the irrigated and rainfed ecosystems. To overcome these conditions, breeding aspects need to focus on developing multiple-stress-tolerant cultivars (MSTC) and improving nutrient use efficiency, which are vital for the sustainability of grain yield productivity, which could have a more significant impact on higher yield, especially under low-fertilizer-input conditions, besides being more beneficial to poor farmers. Several critical components of morphological, physiological, and agronomic traits are reducing grain yield and decreasing biomass content under nutrient-deficient conditions. These traits become altered due to changes in their molecular mechanism and physiological pathways, leading to their susceptibility [22]. The exploitation of rice genetic resources, using advanced genomic technologies, is essential for the identification of rice cultivars with NuUE for increasing crop grain yield under low-input conditions.

Developing rice varieties with stress tolerance and NuUE through conventional breeding approaches is extremely slow because of several factors that influence the molecular genetics and physiological mechanisms underlying low-input tolerance, the complexity of NuUE traits, and the absence of efficient breeding selection criteria [23,24,25]. The combination of advanced molecular marker-assisted breeding and conventional breeding platforms could speed up the breeding procedure for varietal development and identifying trait-associated QTLs. Over the past two decades, several types of traditional molecular markers, such as restriction fragment length polymorphism (RFLP), randomly amplified polymorphic DNA (RAPD), amplified fragment length polymorphism (AFLP) and simple sequence repeats (SSRs), have been used for QTL identification for NuUE-related traits under low-input conditions of nitrogen [26,27,28,29,30], phosphorus [7,31,32,33,34,35,36,37,38], and potassium [39,40]. However, these molecular markers have some disadvantages vis-à-vis SNP markers, such as partial chromosome coverage, labor-intensiveness, low resolution, and higher cost. Among these various molecular markers, SNP markers have a comprehensive range of applications in the construction of genetic maps, uniform distribution, and cloning of QTLs. Their clear advantages, such as high density and accurate and reliable approaches, have been used to perform high-throughput genotyping based on SNPs across the genome [41,42,43,44]. In a genomics era, high-throughput genotyping with next-generation sequencing (NGS) and various array-based SNP detection platforms have become excellent tools for the dissecting of complex traits and identification of trait-associated genes and alleles in rice [45,46,47,48]. As compared to NGS, a SNP array can be used for many samples within a short period; it has a low cost and analysis of data interpretation is relatively easy [47,49]. The recently developed SNP array has been successfully used in diversity studies and genome-wide association studies (GWAS), and in the identification of numerous QTLs and genes in rice [43,49,50,51].

Over the years, the identification of QTLs for varied agro-morphological traits related to NuUE, by using different populations, hardly resulted in any significant impact regarding rice crop improvement and the development of varieties. The QTLs were identified related to NPK use efficiency using recombinant inbred lines (RILs) [34,48,52,53,54], backcross inbred lines (BILs) [55], doubled haploids (DHs) [39,56,57,58,59], BC_2_F_3_ [33], introgression lines (ILs) [60,61], and chromosome segment substitution lines (CSSLs) [35] populations. These QTLs have a smaller effect, and many of them exhibit significant epistatic and QTL x Environment interactions, making them less amenable to breeding programs.

Several QTLs may correspond to known genes in the N or P metabolic pathway, for example, Qyd-2b for N deficiency tolerance was located in the vicinity of the gene encoding cytosolic glutamine synthetase (GS1) [62], and Qyd-3b and Qpn-3 were nearby the genes for glutamate dehydrogenase (GDH2) [26]. Interestingly, Qyd-12 was detected only in low-P conditions and was co-localized with a major QTL, Pup1, on chromosome 12, which was reported to be involved in P absorption [63]. These results indicate that the QTLs specially detected under single N or P deficiency conditions may be involved in different pathways of N and P metabolism. The tightly linked markers have breeding potential in pyramiding elite genes and QTLs for N and P use efficiency.

Although N and P use efficient QTLs and linked traits were reported earlier in different genetic backgrounds of mapping populations, to date, there are no reports of different combinations of NPK fertilizers and their response to the foremost NuUE traits, such as grain yield response, agronomic efficiency, and partial factor productivity in rice. In the present study, taking advantage of the rapid development of SNP technologies, we used a 6K SNP array to genotype a BC_1_F_5_ introgression line (IL) derived from a cross between Weed Tolerant Rice 1 (WTR-1), as the recipient parent, and Hao-an-nong (HAN), as the donor parent. In this study, we identified the NuUE-related QTLs by subjecting the population to varying nutrient rates and understanding the performance of yield and its components.

## 2. Results

### 2.1. Phenotypic Variation of NuUE Traits and Their Correlation among Traits

**Figure 1:**
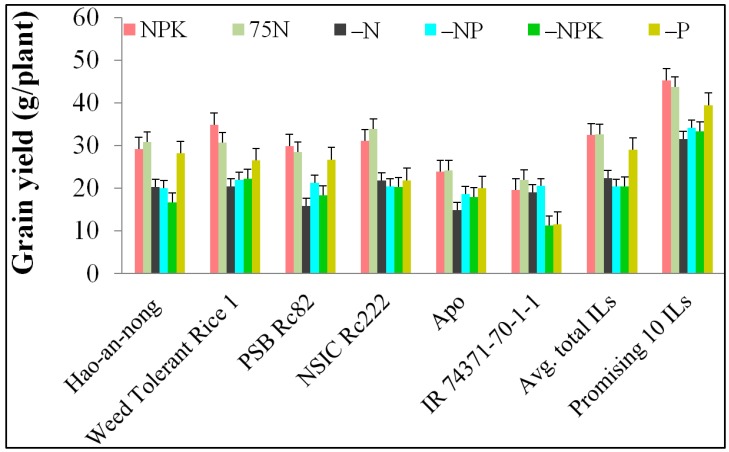
Grain yield performances of introgression lines, parents and checks in all six nutrient conditions.

A total of 230 BC_1_F_5_ ILs were evaluated in six different NPK combinations (–N, –P, –NP, –NPK, 75N and NPK) during the dry season of 2014 in the experimental fields of International Rice Research Institute (IRRI). The key traits of NuUE were analyzed in the total of ILs and were compared with those of the parents and checks (Figure 1). The descriptive statistics for five traits as grain yield (GY), 1000-grain weight (1000-Gwt), percentage of spikelet fertility (PSPF), biomass yield (BY), filled grains per plant (FGN) mean values and testing of significance are presented in Table 1 and Table 2. The highest GY range of 19.29–50.26 g/plant was observed in NPK, followed by 20.82–48.83 g/plant in 75N, and 12.81–42.95 g/plant in –NPK, and the lowest GY range of 13.89–34.96 g/plant was detected in –N conditions. Among these six nutrient conditions, 18 ILs in NPK, 23 ILs in 75N, 4 ILs in –P, and 1 IL in both –NPK and –NP had more than 40 g/plant. In –N conditions, the lowest GY was observed (34.96 g/plant). The best performing ILs under each of the six nutrient conditions with the highest grain yields were *GSR IR2-1-RF6-NU7-NU2-NU76-NU96* -WTR 1-RF6 in NPK that gave 50.26 g/plant; and 42.95 g/plant (*GSR IR2-1-RF6-NU7-NU3-NU82-NU97*-WTR 1-RF6) in –NPK; 48.83 g/plant (*GSR IR2-1-Y17-NU2-NU5-NU6-NU9*-WTR 1-Y17) in 75N; 34.96 g/plant (*GSR IR2-1-L3-NU1-NU1-NU1-NU1*-WTR 1-LI3) in –N; 40.94 g/plant (*GSR IR2-1-RF6-NU4-NU9-NU14-NU66*-WTR 1-RF6) in –P; and 40.43 g/plant (*GSR IR2-1-RF6-NU7-NU2-NU77-NU94*-WTR 1-RF6) in –NP conditions, respectively. Across all six nutrient conditions, the lowest GYs were observed in WTR 1-RF13 (*GSR IR2-1-RF13-NU2-NU8-NU4-NU12*), WTR 1-RF14 (*GSR IR2-1-RF14-NU2-NU4-NU6-NU3*) with 11.85 g/plant; 12.43 g/plant detected in –NP; and followed by WTR 1-LI3 (*GSR IR2-1-L3-NU1-NU1-NU3-NU5*) and WTR 1-LI12 (*GSR IR2-1-L12-NU1-NU4-NU7-NU5*) with 12.86 g/plant and 12.81 g/plant in –NPK conditions, respectively. Under –NP and –NPK conditions, the recipient parent (Weed Tolerant Rice 1) gave 19.87 g/plant and 20.94 g/plant, respectively, while the donor parent (Hao-an-nong) gave 15.73 g/plant and 16.02 g/plant, respectively.

After further analysis of ILs with higher grain yield in each NuUE condition, we selected the first ten highest GY lines, and GY ranged from 28.86 to 50.26 g/plant. Interestingly, we identified promising lines, WTR 1-RF6 (*GSR IR2-1-RF6-NU7-NU3-NU82-NU97*), that showed higher GY in four different nutrient conditions: –N (29.11 g/plant), –P (38.40 g/plant), –NP (31.90 g/plant), and –NPK (42.95 g/plant). Similarly, introgression line WTR 1-RF6 (*GSR IR2-1-RF6-NU7-NU2-NU77-NU94*) had higher GY in three different NuUE conditions, –NP (40.43 g/plant), –NPK (31.43 g/plant), and NPK (42.76 g/plant); and WTR 1-RF6 (*GSR IR2-1-RF6-NU7-NU2-NU76-NU96*) in –P (40.09 g/plant), –NP (33.09 g/plant), and NPK (50.26 g/plant) conditions. Likewise, WTR 1-Y17 (*GSR IR2-1-Y17-NU2-NU5-NU6-NU8* and *GSR IR2-1-Y17-NU2-NU5-NU6-NU9*) had higher GY in 75N (42.00 g/plant and 48.83 g/plant, respectively), –NP (35.73 g/plant and 33.41 g/plant, respectively), and –NPK (33.29 g/plant and 34.75 g/plant, respectively) conditions, The results for NuUE key traits, such as GY (–NP, –NPK), FGN (75N, –NP, –NPK), and BY (NPK, 75N, –N, –P, –NP, –NPK), had a high level of phenotypic variation with a coefficient of variation (CV) of above 20%. The correlations of five traits in six different nutrient conditions were identified to show significant relationships between the respective traits. GY was found to be significantly and positively correlated with BY; FGN in NPK; FGN in 75N; GWT, BY, and FGN in –P; PSPF, BY, and FGN in –N; and BY and FGN in –NP (*p* < 0.01) conditions. However, GY showed itself to be negatively correlated with 1000-Gwt in NPK and with BY in 75N conditions. Among the six nutrient conditions, GY was negatively correlated with BY under 75N. However, the remaining GWT, PSPF and FGN traits followed with significant positive correlation between the traits.

### 2.2. ANOVA and Interaction with the Environment

The fixed effect model was used for the analysis of variance with Satterthwaite denominator, and the summary of ANOVA is given in Table 2. Among the six nutrient conditions, significant genotypic effects were observed under –N (*F* = 1.44, *p* = 0.0005), –NP (*F* = 1.76, *p* < 0.0001), and –NPK (*F* = 2.47, *p* = 0.0007) conditions at the 1% level. In contrast, under 75N (*F* = 1.2, *p* = 0.053), –P (*F* = 1.38, *p* = 0.0658), and NPK (*F* = 1.07, *p* = 0.4293) conditions, the genotypes were not significantly different from each other. It was noted that the treatments showing significant genotypic effects are those that lack the nitrogen fertilizer component, indicating that some of these materials are nutrient use efficient. In the significant effect of fertilizers with genotype, the study used a −2 log-likelihood ratio test and found a significant environmental effect at the 1% level (*χ*^2^ = 15.23, *p* = 0.0001). However, genotype and environment showed non-significant interactions (*χ*^2^ = 0.00, *p* = 0.9992) (Table 3). This suggests that the different fertilizer applications significantly affect yield performance of the ILs as a whole. On the other hand, the non-significant genotypes by environment interactions indicate the consistent performance of genotypes across environments.

### 2.3. Analysis of Agronomic Efficiency (AE) and Partial Factor Productivity (PFP)

In the NuUE study, agronomic use efficiency and partial factor productivity were calculated using the fertilizer application rate of NPK (160–50–50 kg ha^−1^). The computation using the equations (i to iii) revealed 15.06–34.11 kg of grain for each kg of N derived from an NPK fertilizer given in 3.2:1:1 ratio, respectively. However, using this equation, we found 117 ILs with >15 kg of grain per kg^−1^ N (NPK), along with 33 ILs (–P) and 161 ILs (75N). These were also compared with parents and we identified 42 ILs, 23 ILs, and 79 ILs that had a higher AE with respect to each equation (Table 4). Similarly, analysis of partial factor productivity helped to identify 16 ILs (NPK), 4 ILs (–P), and 151 ILs (75N) that had a >50 kg of grain per kg^−1^ N (NPK) condition. In comparison with parents, 25 ILs (NPK), 61 ILs (–P), and 117 ILs (75N) showed more than 50 kg of grain per kg^−1^ N in each condition.

### 2.4. Construction of Linkage Map and Segregation of SNP Markers

**Figure 2:**
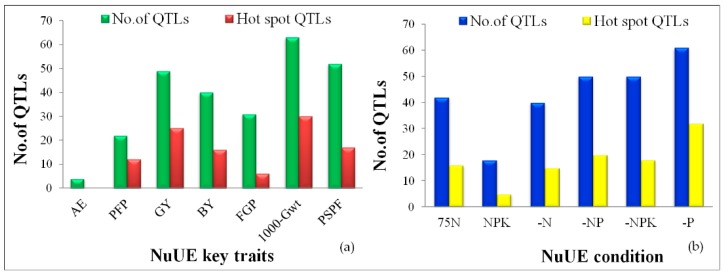
Distribution of trait-wise (**a**); and NPK combinations (**b**), associated with a total number of quantitative trait loci (QTLs) and hotspot QTLs in different nutrient conditions in rice.

**Figure 3:**
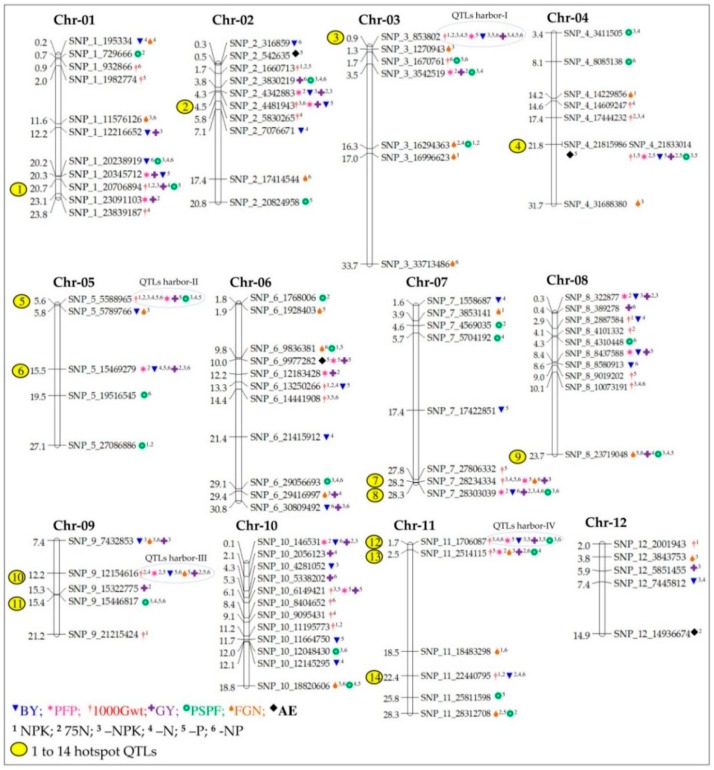
Linkage map of 261 QTLs distributed on 12 chromosomes, with respective polymorphic markers and colors depicting the QTLs governing crucial different nutrient traits.

A total of 704 high-quality polymorphic SNP markers, with an average for each chromosome of 58 SNP markers, were used to genotype the 230 ILs, and a high-density genetic linkage map was developed covering 1526.8 cM, with an average of 127.2 cM per SNP marker (Table 5). The logarithm of the odds (LOD) thresholds and explained phenotypic variance for six nutrient conditions ranged from 2.52% to 17.76% and 5.87% to 34.68%, respectively. Using single marker analysis (SMA), a total of 261 QTLs were mapped on all 12 chromosomes, with an average of 21 QTLs for yield attributed to seven key component traits of NuUE in rice. Among the 12 chromosomes, the highest number of QTLs was located on chromosome 6 (26 QTLs), whereas the lowest number of QTLs was observed on chromosome 12 (6 QTLs), and all QTLs are listed in Table S1. The hotspot QTL regions are shown in Figure 2 and Figure 3. The majority of the QTLs were associated with 1000-Gwt, and PSPF traits had a negative additive effect of 61.9% and 61.5%, indicating that alleles from the recipient parent WTR-1 contributed to increasing phenotype.

Out of the 261 QTLs identified, 62.5% (163 QTLs) had a positive additive effect contributed by WTR-1, and the remaining 37.5% followed with the negative allele from donor parent Hao-an-nong. QTLs with >20% phenotypic variation explained (PVE) were determined as major QTLs with an LOD score of >9.50, while others were considered as minor QTLs. In summary, a total of 261 QTLs for seven traits under six different combinations of NPK nutrients were identified, out of which 49 were major QTLs and 212 were minor QTLs (Table S1). Out of these NuUE conditions, a large number of QTLs were observed in –P (61 QTLs), –NP, and –NPK (50 QTLs), and followed with 75N (42 QTLs) and –N (40 QTLs), and the fewest were detected in NPK conditions with 18 QTLs. The PVE of significant major QTLs ranged from 20.25% to 34.68% at 9.55 to 17.76 LOD value, whereas, for minor QTLs, the explained PV ranged from 5.87% to 19.98% at 2.52 to 9.49 LOD value. The distribution of all major and minor QTLs, represented on all 12 chromosomes, is shown in Figure 3, and the hotspot QTLs (>5 QTLs), identified over nine chromosomes (chr01, chr02, chr03, chr04, chr05, chr07, chr08, chr09, and chr11), were linked to six essential nutrient traits (PFP, GY, BY, FGP, 1000-Gwt, and PSPF) in rice.

Out of the 704 polymorphic markers, SNP_2_4481943 had the highest LOD value recorded (17.76), which explained PV of 34.68%, followed by two other markers, SNP_10_6149421 (LOD 16.28) and SNP_8_10073191 (LOD 15.31), which explained PV of 32.32% and 30.73%, respectively. Interestingly, these QTLs were significantly associated with 1000-Gwt under —NPK conditions, and their negative value of the allele was exhibited from donor parent Hao-an-nong. In contrast, the lowest LOD (2.52) was recorded in SNP_6_9836381, which explained PV of 5.87%, followed by three other SNP markers, SNP_12_14936674 (2.55), SNP_12_7445812 (2.55), and SNP_12_5851455 (2.53), which explained PV of 5.92%, 5.92%, and 5.89%, respectively. These four QTLs were linked with different key traits such as FGN, AE, BY, and GY under –NP, 75N, –N, and –NPK conditions. The lowest LOD value of two markers (SNP_6_9836381 and SNP_12_14936674) was contributed by the negative allele from donor parent Hao-an-nong, and two other markers (SNP_12_7445812 and SNP_12_5851455) had a positive allele from recipient parent WTR-1.

### 2.5. QTLs for NuUE Traits

#### 2.5.1. Agronomic Efficiency (AE)

Agronomic efficiency (AE) was determined in the experiment on NuUE, and we found four QTLs (*qAE_2.1*, *qAE_4.1*, *qAE_6.1* and *qAE_12.1*) that were located on chromosomes 2, 4, 6, and 12, respectively (Table 6). For the agronomic efficiency of applied nitrogen in terms of –P conditions (applied nitrogen and potassium fertilizer), three QTLs were detected with an LOD score and phenotypic variation of 2.77, 4.01, and 4.52 and 6.43%, 9.17%, and 10.27%, respectively, with a positive additive effect of 3.16, 2.13, and 2.28, which indicates that the progeny carried the trait from the recipient parent WTR-1. QTL *qAE_6.1* consisted of a peak marker (SNP_6_9977282) that was also associated with GY and PFP under –P conditions, with explained phenotypic variation of 17.6%. In 75N conditions, one QTL (*qAE_12.1*) was detected with a peak marker (SNP_12_14936674) located on 14936674 bp of chromosome 12, whereas LOD score and phenotypic variation were 2.55% and 5.92%, respectively, since the additive value of –2.8 shows that the trait in the progeny came from donor parent Hao-an-nong.

#### 2.5.2. Partial Factor Productivity (PFP)

A total of 22 QTLs were identified across all chromosomes, except chromosome 12, under two different nutrient conditions (–P and 75N). Eleven QTLs were associated with PFP with LOD scores ranging from 3.39 (SNP_5_5588965) to 12.15 (SNP_10_6149421), which explained phenotypic variation from 7.81% to 25.28% in –P conditions. Among these 11 QTLs, the highest LOD value of three peak markers, SNP_2_4481943 (LOD 11.68), SNP_8_8437588 (LOD 9.9), and SNP_10_6149421 (LOD 12.15), was contributed by the Hao-an-nong allele on chromosomes 2, 8, and 10, and eight QTLs remained on chromosomes 1, 3, 4, 5, 6, 7, 9, and 11 that had a positive additive value, indicating the contribution of recipient parent WTR-1. Under –P conditions, one of the peak markers (SNP_1_20345712) was located at the 20345712 bp position on chromosome 1, which is associated with BY and GY, with an LOD score of 4.01 and 8.63, explaining a PV of 9.17% and 18.71%, respectively (Table 6). Similarly, we discovered 11 significant QTLs for PFP under 75N conditions. Of the 11 QTLs detected, four (*qPFP_2.1*, *qPFP_5.2*, *qPFP_8.1*, and *qPFP_10.1*) were contributed by the donor parent allele of Hao-an-nong, and the remaining seven QTLs by the WTR-1 allele. An average LOD value of 7.22 and PV of 15.82% were explained by 11 QTLs. The two highest peak markers were SNP_5_15469279 and SNP_2_4342883, located on chromosomes 2 and 5, respectively, which explained PV of 20.25% and 20.91%.

#### 2.5.3. Grain Yield (GY)

Forty-nine QTLs were identified for GY. Each QTL explained 5.89%~25.28% of the phenotypic variation, with respective LOD scores of 2.53~12.15. Among the total QTLs of GY, nine QTLs showed more than 20% of the phenotypic variance and were located on chromosome 2, 4, 5, 8, and 10. Four GY QTLs (*qGY_2.4*, *qGY_4.2*, *qGY_8.4*, and *qGY_10.5*) were detected in –P, two QTLs (*qGY_5.4*, *qGY_10.4*) in –NP and one QTL (*qGY_5.3*) significantly expressed under –NPK condition. Similarly, two GY QTLs (*qGY_2.2* and *qGY_5.2*) were identified in 75N nutrient condition. The peak marker of SNP_5_15469279 was located on chromosome 5, and it was associated with GY under three different nutrient conditions, such as 75N, –NPK, and –NP. Whereas LOD and phenotypic variations of this marker was recorded 9.78, 9.97, and 9.92 and 20.91%, 21.26%, and 21.18%, respectively, since additive value shows negative allele contributing from a donor parent Hao-an-nong.

#### 2.5.4. Biomass Yield (BY)

A total of 40 QTLs were discovered in six nutrient conditions, except for NPK. Under deficiency of –N, –P, –NP, and –NPK conditions, 39 QTLs were linked with BY on all 12 chromosomes. Chromosomes 1, 2, 5, 8, 10, and 11 had more than three QTLs controlling BY. The contributions of these QTLs showed phenotypic variation, ranging from 5.99% to 17.63%, and their LOD values ranged from 2.57 to 8.08, respectively. These QTLs had the largest effects, with negative additive values showing them to be contributed by donor parent Hao-an-nong to the progeny. Under 75N conditions, one QTL (*qBY_11.3*), located on chromosome 11, at the position of 22440795 bp, had a PVE of 7.37% and it was contributed by the Hao-an-nong allele.

#### 2.5.5. Percentage of Spikelet Fertility (PSPF)

In total, 52 QTLs were identified for six nutrient conditions and they were distributed on all chromosomes except for chromosome 12. These QTLs explained phenotypic variation ranging from 5.98% to 29.76%. Of these 52 QTLs, 6 QTLs for 75N, 3 QTLs for NPK, 10 QTLs for –P, and 11 QTLs for each nutrient condition (–N, –NP, and –NPK) were significantly expressed, with an LOD score ranging from 2.57 to 14.73. Under –N deficiency conditions, four QTLs (*qPSPF_3.3*, *qPSPF_6.4*, *qPSPF_8.2,* and *qPSPF_9.1*) and another three QTLs (*qPSPF_3.4*, *qPSPF_6.5,* and *qPSPF_9.2*) for —NPK conditions were recorded with PVE of >20%. The peak markers were located on chromosomes 3, 6, 8, and 9 with the favorable allele contribution from WTR-1.

#### 2.5.6. 1000-Gwt

Out of the 261 QTLs, 63 were identified in 1000-Gwt for six nutrient (–N, –P, –NP, –NPK, 75N, and NPK) conditions. These QTLs together explained an average phenotypic variation ranging from 5.95% to 34.68% and an LOD score of 2.56 to 17.76. Among these QTLs, 24 (38%) were from donor parent Hao-an-nong, which was lower than WTR-1’s contribution with 39 QTLs (62%). The QTLs *q1000Gwt_2.4*, *q1000Gwt_8.5,* and *q1000Gwt_10.1* had an LOD score greater than the threshold of 15 and explained PV of 17.76%, 15.31%, and 16.26%, respectively, under –NPK conditions. The three highest LOD values (17.76, 15.31, and 16.28) were observed on chromosomes 2, 8, and 10 and were contributed by the WTR-1 allele

#### 2.5.7. Filled Grain Number (FGN)

A total of 31 QTLs had an LOD score of 2.52 to 7.59 (higher than the threshold level) and were located across all 12 chromosomes identified under six different nutrient conditions. The QTLs had phenotypic variation ranging from 5.87% to 15.84%. The QTLs *qFGN_4.2*, *qFGN_5.1,* and *qFGN_7.1* on chromosomes 4, 5, and 7 had a large additive effect (−118.1, −127.97, and −139.93), accounting for 7.16%, 10.17%, and 7.25% of the phenotypic variation, respectively. The alleles for increasing FGN came from both Hao-an-nong (10 QTLs) and WTR-1 (21 QTLs). Only two QTLs (*qFGN_1.1* and *qFGN_3.4*) controlling FGN on chromosomes 1 and 3 were detected, and explained 10.23% and 8.82% of the phenotypic variation under –N conditions, respectively. Out of 31 QTLs, 10 were contributed with an allele from Hao-an-nong, and the remaining 21 QTLs carried an allele from recipient parent WTR-1.

#### 2.5.8. Hotspot QTLs for Multiple Traits

Among the total of 261 QTLs, 106 were identified as promising QTLs located over 14 hotspot regions of nine chromosomes (1, 2, 3, 4, 5, 7, 8, 9, and 11), and each chromosome had more than five QTLs contributing to single SNP peak markers (Figure 2a,b). The QTLs identified under each of the six nutrient conditions showed 32 QTLs identified in –P conditions, and these were significantly linked with six traits (PFP, GY, BY, FGP, 1000-Gwt, and PSPF) that explained PV ranging from 5.99% to 27.9% with LOD values of 2.57 to 13.64. Subsequently, several QTLs were identified under five nutrient conditions, –N (15 QTLs), –NP (20 QTLs), –NPK (18 QTLs), 75N (16 QTLs), and NPK (5 QTLs), and these were discovered across nine chromosomes. On each chromosome, the hotspot regions were identified that contributed to different traits under six different nutrient conditions. The peak marker SNP_1_20706894 on chromosome 1 was found to be linked to five QTLs associated with three traits, 1000-Gwt (75N, NPK, and –NPK), GY (–N), and PSPF (–P) conditions. Similarly, five QTLs located on chromosomes 2, 9, and 11, with peak markers SNP_2_4481943, SNP_9_15446817, and SNP_11_2514115, respectively, were associated with five traits: 1000-Gwt, PFP, BY, GY, and PSPF. Further analysis of the remaining hotspot regions, chr04 (eight QTLs), chr05 (seven QTLs), chr07 (eight QTLs-SNP_7_28303039 and six QTLs-SNP_7_28234334), chr08 (six QTLs) and chr11 (six QTLs), showed association with six prominent critical traits: 1000-Gwt, PFP, BY, GY, PSPF, and FGN. One to 14 hotspot QTLs per chromosome were classified (Table S1 and Figure 3) for the discovery of the top hotspot QTLs (a QTL that contributes to ≥10 QTLs). Out of these 14 hotspot QTLs, four QTLs were located on chromosome 3 (harbor-I QTLs), 11 QTLs on chromosome 5 (harbor-II QTLs), and 10 QTLs on chromosome 9 (harbor-III QTLs) and chromosome 11 (harbor-IV QTLs) at the nucleotide positions of 853,802; 5,588,965; 12,154,616; and 1,706,087 bp, respectively. The top hotspot regions of each chromosome were designated as QTLs harbor-I to -IV. While analyzing these top QTLs, harbor-I to -IV, we identified a positive allele contributed from recipient parent WTR-1 (Figure 3). Of these harbor-I to -IV QTLs, 16 QTLs were expressed on chr03, chr05, chr09, and chr11 for –P; nine QTLs on chr03, chr05, and chr11 for –NPK; seven QTLs on chr03, chr05, chr09, and chr11 for –NP; six QTLs on chr03, chr09, and chr11 for –N; five QTLs on chr03, chr05, and chr09 for 75N, and two QTLs on chr03 and chr05 for NPK, respectively. As a result, the top four hotspot QTLs explained phenotypic variation ranging from 6.09% to 26.97% and LOD scores ranging from 2.62 to 13.11.

#### 2.5.9. Fine-Tuning of QTLs Harbor-I to -IV

The top four hotspot QTLs of chromosomes 3, 5, 9, and 11 were refined through QTARO databases. The first QTL (harbor-I) was identified on chromosome 3, between peak markers SNP_3_853802 and SNP_3_16294363, covering a span of 15.44 Mbp regions. In this region, nine genes (*pez1*, *OsIRO3*, *Mit*, *OsApx1*, *RPN10*, *OsFRDL1*, *OsMTP8.1*, *OsGS1*; *2,* and *OsPT2*) and four QTLs (*qRFWw3*, *n-p3*, *qDLR3,* and *qZNT-3*), documented based on earlier reports, were associated with soil stress tolerance mechanism-related traits, for uptake and translocation from roots to shoots, for various nutrients, such as iron, cadmium, phosphate, and manganese (Table S2). Two transcription factors (*IDEF2* and *OsHsfA4a*) and one gene (*OsZIP5*), identified in the region spanning 21.5 Mbp on chromosome 5 (QTL harbor-II); two QTLs (*qLBI-9* and *qALSRL-9*) and one gene (*OsSTR1*), located on chromosome 9 covering a 13.78 Mbp region (QTL harbor-III); and two QTLs (*qDLR11* and *qRRE-11*), identified on chromosome 11 covering a region of 26.62 Mbp (QTL harbor-IV), were identified. Of these eight QTLs, QTL harbors distributed on three chromosomes (3, 9, and 11) accounted for PV ranging from 3.47% (*qDLR3*) to 18.3% (*qALSRL-9*) and LOD value ranging from 2.64 (*qRRE-11*) to 15.23 (*qn-p3*).

## 3. Discussion

NuUE is a complex trait influenced by several factors, and it is considered a vital trait to improve rice grain yield productivity in marginal and rainfed lowland areas. Rice breeding programs need to incorporate NuUE traits as this helps resource-poor farmers to save on fertilizer, and allows maximization of valuable resources to increase profitability. To date, several morphological and agronomic traits have been identified, and they can be used as indicators of nitrogen (N) [52,64,65,66], phosphorus (P) [7,38,67,68,69,70], and potassium (K) [16,39,40,71,72,73] deficiency tolerance under both field and hydroponic conditions. Using different genetic backgrounds of mapping populations, such as RILs, NILs, BILs, ILs, CSSLs, DHs, and BC_2_F_4_, there are several reports on QTL identification with independent studies of low-N, -P, and -K conditions. The previously identified QTLs were mapped on different chromosomes using low-density linkage maps that were constructed by PCR-based molecular markers, such as RFLP, AFLP, SSR, and STS [26,29,31,34,39,48,52,55,58,67,74,75,76]. However, in this study, we used SNP markers to identify the QTLs.

For the identification of QTLs, single-marker analysis, simple interval mapping, and composite interval mapping are widely used methods [77,78]. The development of nutrient use efficient selective ILs, through an early generation backcross breeding approach, allows both the detection of QTLs for NuUE traits and the simultaneous development of promising breeding materials into varieties. This is the strength of selective introgression breeding first proposed by Tanksley [78], and later demonstrated for rice by Li et al. [79]. However, for QTL detection in selective introgression backcross breeding populations, we could use single marker analysis (SMA) and association mapping with single nucleotide polymorphism (SNP) markers, and use a higher threshold of LOD value of 2.5 to declare a strong QTL.

### 3.1. Analysis of Critical NuUE Traits

The phenotypic evaluation of 230 BC_1_F_5_ ILs varied significantly in six different NPK nutrient combinations: N, –P, –NP, –NPK, 75N, and NPK. Grain yield is a quantitative trait and a highly complex character for all crops [80], and is the essence of any breeding program. Various morphological and physiological plant traits contribute to grain yield. Yield components are inter-related with each other, indicating a complex chain of relationships, which is highly influenced by the environment [81]. The breeding strategy in rice mainly depends on the degree of associated characters as well as the magnitude and nature of variation [82,83]. Based on a higher grain yield level, 18 ILs for NPK, 23 ILs for 75N, 4 ILs for –P, and 1 IL for both –NPK and –NP had more than 40 g/plant, and in –N conditions the lowest GY was observed (34.96 g/plant).

An understanding of the correlations between traits is of great importance in breeding programs, especially if the selection of one of them is impaired by low heritability or difficulties of measurement and identification [84]. Information on trait correlations has been helpful as a basis for selection in breeding programs. Ashura [85] showed a positive correlation between the number of filled grains per panicle, number of panicles per plant, and 1000-grain weight for grain yield. A correlation study enables breeders to understand the major traits for which selection can be based on population improvement. GY had a significant positive correlation with BY and FGN in NPK; FGN in 75N; GWT, BY, and FGN in –P; PSPF, BY, and FGN in –N; and BY and FGN in –NP (*p* < 0.01) conditions. A negative correlation was recorded for GY with 1000-Gwt in NPK and for BY in 75N conditions. Under all six nutrient conditions, GY showed a negative relationship with BY and the remaining traits, GWT, PSPF, and FGN, had a significant positive correlation between the traits. Highly correlated traits were indicated to have a common genetic basis, suggesting that these eight relative phenotypic traits could be used for the evaluation of P-deficiency tolerance in BILs at the seedling stage [86].

### 3.2. Promising Traits of AE-Associated QTLs

The application of AE and PFP traits was essential to plant nutrients for an optimum quantity and right proportion, through the correct method and time of application, and this is the key to increased and sustained crop production [87]. These two traits are important for the measurement of nutrient use efficiency, and this also provides an integrative index that quantifies total economic output relative to the use of all the nutrient resources in the system [88]. In 1996, Cassman et al. [10] defined PFP and AE and showed that they could be increased by raising the amount, uptake, and use of available nutrients, and by increasing the efficiency with which applied nutrients are taken up by the crop and used to produce grain. In this study, we identified 42 ILs with higher AE in NPK, 23 ILs in –P, and 79 ILs in 75N, as compared with their parents, and they were explicit in >15 kg grain kg^−1^ N conditions. Yoshida [89] and Cassman et al. [10], respectively, estimated AE to be 15–25 kg kg^−1^ and 15–20 kg kg^−1^ in the dry season in farmers’ fields in the Philippines. In a similar way, Wen-Xia et al. [90] mentioned the AE in two kinds of rice: Jinzao had an AE range of 8.02–20.14 kg grain kg^−1^ N and Shanyou63 had an AE range of 3.40–18.37 kg grain kg^−1^, differing with N management. In hybrid rice, AE for applied P was 5.2 kg grain kg^−1^ P and for applied K was 11.8 kg grain kg^−1^ K, where the fertilizer application rate for NPK was 200–75–200 and 200–150–200 kg ha^−1^, respectively; AE for applied P in non-hybrid rice was 2.3 kg grain kg^−1^ P and 4.7 kg grain kg^−1^ P, where the fertilizer rate was the same [81].

Four novel QTLs were identified for AE (*qAE_2.1*, *qAE_4.1*, *qAE_6.1,* and *qAE_12.1*) on chromosomes 2, 4, 6, and 7, which explained phenotypic variation ranging from 5.93% to 10.27%. For these four AE QTLs, each SNP marker position was analyzed at both sides of the 500 kp (up- and down-stream of 1 Mb) regions on the chromosome (Table S3). With the respective positions of the 1 Mb region on chromosome 2, seven genes were identified influencing diverse functional roles as a defense to pathogen resistance and physiological mechanisms. Of the seven genes revealed, *BiP3* for BLB resistance [91]; *OsHPL3* for multiple biotic disease resistance, such as brown planthopper, striped stem borer, and BLB [92]; *BiP1* (*Os06g0622700*) for increased seed storage protein, starch content in the endosperm, and also stress responses [93,94]; *OsHPR1* (*Os02g0104700*) involved in photo-respiratory metabolism [95]; and *OsNOA1* regulating chlorophyll biosynthesis, plastid development, and Rubisco formation in a temperature-dependent manner [95], were identified on chromosome 2. Similarly, on chromosome 6, genes *Pi9* and *Pi2* were noticed [96], which are related to blast disease resistance, along with another three QTLs (*qPHw6-2*, *qSFWd6*, *amy6-1*) related to drought tolerance and amylose content [97,98]. Interestingly, a major QTL for P deficiency tolerance that was previously mapped on chromosome 12 was found to be closely associated with peak marker SNP_12_14936674 [31], and in the same region of markers was linked with drought tolerance QTLs [99].

In PFP analysis, 16 ILs in NPK, 4 ILs in –P, and 151 ILs in 75N conditions yielded >50 kg grain kg^−1^ N and, in comparison with parents, 25 ILs (NPK), 61 ILs (–P), and 117 ILs (75N) yielded more than 50 kg grain kg^−1^ N in each condition. Amanullah et al. [100] showed, with a maize crop, a PFP of applied N of 36.62 kg grain kg^−1^ N and an AE of applied N of 22.49 kg grain kg^−1^ N. After applying DAP and SSP in fields, the AE of two fertilizer applications resulted in 13.01 and 13.71 kg grain kg^−1^ P, and PFP resulted in 63.58 and 61.92 kg grain kg^−1^ P. In this study, 22 QTLs for PFP traits were identified under 75N (11 QTLs) and –P (11 QTLs) conditions, with a PV ranging from 8.41% to 20.91% and 7.81% to 25.28%, respectively. Out of these 22 QTLs, six QTLs at SNP marker positions were analyzed on both sides of 500 kb (up- and down-stream of 1Mb) regions, which were explained by PV of more than 20% on chromosomes 2, 4, 5, 8, and 10. Five genes and six QTLs were identified in the 1 Mb region associated with PFP traits. On chromosome 2, three genes were identified as *OsWRKY71* [101], *Os4CL3* [102], and *OsWRKY45* [103,104], which were functionally related to defense signaling molecules, such as SA, Me, JA and lignin biosynthesis. Two QTLs (*qDSR_8* and *qALRR_8*) on chromosome 8 [105,106] and three QTLs (*qRRE_10*, *qRFW_10,* and *qRCCL_10*) [107,108] and one gene, *OsAT1/Spl18* [109], located on chromosome 10 revealed having tolerance of aluminum and alkaline stress, and also showed blast disease and ultraviolet-B resistance in rice. On chromosome 5, gene *OsWRKY45* was found to be closely associated with SNP_5_15469279, and it is involved in multiple biotic and abiotic stress tolerance/resistance, such as BLB, blast, sheath blight, drought, salinity, and cold [103,104,110].

### 3.3. Categorizing NuUE QTLs and Related Traits

Averages of 21.75 QTLs were distributed across all 12 chromosomes. Out of 261 QTLs, the highest numbers were located on chromosome 3 (28 QTLs), chromosome 11 (27 QTLs), chromosome 6 (26 QTLs), chromosome 1 (24 QTLs), and chromosomes 5, 8, and 10 (23 QTLs), and the remaining chromosomes 2, 4, 7, and 9 had a range of 18 to 22 QTLs. All the identified QTLs had PVE ranging from 5.87% to 34.68% and an LOD threshold range of 2.52–17.76. The lowest number of QTLs was observed on chromosome 12, with PVE from 5.89% to 9.49% and LOD value of 2.6 to 11.82. Based on the different nutrient conditions, the largest number of QTLs was detected under P deficiency (–P: 61 QTLs), and the lowest number of QTLs was identified for NPK (18 QTLs) conditions. For other nutrient conditions, the numbers were as follows: –NP (50 QTLs), –NPK (50 QTLs), –N (40 QTLs), and 75N (42 QTLs). A comprehensive literature survey revealed that a majority of the QTLs associated with low phosphorus were reported on chromosomes 1, 2, and 12 (recently reviewed by Mahender et al. [7], Ali et al. [20], and van de Wiel et al. [70], whereas, for low nitrogen conditions, the majority of morpho-physiological trait-linked QTLs in rice were located on chromosomes 3, 5, and 8 [34,52,53,54,76]. Under six different nutrient conditions, 24.1% of the QTLs were associated with 1000-Gwt, followed by 19.9% for PSPF, 18.7% for GY, 15.3% for BY, 11.8% for FGP, 8.4% for PFP, and 1.5% for AE (Figure 3).

### 3.4. Consistency and Comparisons of Major QTLs across Different Genetic Backgrounds

In this study, a total of 261 QTLs were identified using a 6K SNP array-based genetic linkage map analysis, in 230 BC_1_F_5_ introgression lines, that were tested under six different nutrient conditions. Out of 261 QTLs, 49 major QTLs were found with more than 20% PVE and LOD threshold value ranging from 9.44 to 17.76 distributed across all chromosomes, except for chromosomes 7, 11, and 12. Of these 49 QTLs, the highest number (27 QTLs) was associated with 1000-Gwt and the lowest number (6 QTLs) with PFP. For other traits, nine QTLs were associated with GY and seven QTLs with PSPF, which were detected across five nutrient conditions: 75N, –P, –N, –NP, and –NPK. Interestingly, out of these 261 QTLs, 14 QTLs were consistently detected under more than four nutrient treatments, indicating that these were critical traits that were controlled by similar genes under different nutrient conditions. The consistencies of QTLs were identified on nine chromosomes: 1, 2, 3, 5, 7, 8, 9, 10, and 11.

On chromosome 2, SNP_2_4342883 was linked with four QTLs (*qPFP*, *qBY*, *qGY,* and *q1000Gwt*), and SNP_5_15469279 was linked with three QTLs (*qPFP*, *qBY*, and *qGY*) on chromosome 5, which were expressed under four nutrient conditions: –P, –NP, –NPK, and 75N. On chromosome 7, SNP_7_28303039 was linked with four QTLs (*qPFP*, *qBY*, *qGY,* and *qPSPF*) and, at another location on the same chromosome, SNP_7_28234334 was associated with four QTLs (*q1000gwt*, *qPFP*, *qFGN,* and *qGY*) that were identified in –N, –NP, –NPK, and 75N conditions. In a similar way, on chromosome 8, SNP_8_23719048 was linked with three QTLs (*qFGN*, *qGY*, and *qPSPF*); on chromosome 10, SNP_10_18820606 was linked with two QTLs (*qFGN* and *qPSPF*); on chromosome 11 with six QTLs (*q1000gwt*, *qPFP qBY*, *qGY*, *qPSPF*, and *qFGN*); and, on chromosome 9, SNP_9_12154616 was linked with four QTLs (*q1000gwt*, *qPFP*, *qBY*, and *qFGN*), which were consistently recorded in all nutrient deficiency conditions, such as –N, –P, –NP, and –NPK, respectively. Under –N, –P, –NPK, and 75N conditions, on chromosome 1 (SNP_1_20706894), three QTLs (*q1000gwt*, *qGY,* and *qPSPF*) were identified. Further, two QTLs (*q1000gwt*, and *qBY*) on chromosome 11 (SNP_11_22440795) and five QTLs (*q1000gwt*, *qPFP*, *qGY*, *qPSPF,* and *qFGN*) on the same chromosome were consistently expressed in –N, –P, –NP, –NPK, and 75N conditions. However, five QTLs (*q1000Gwt*, *qPFP*, *qBY*, *qFGN,* and *qGY*) located on chromosome 3 (SNP_3_853802) and four QTLs (*q1000Gwt*, *qPFP*, *qGY,* and *qPSPF*) located on chromosome 5 (SNP_5_5588965) were detected consistently under six NuUE conditions: –N, –P, –NP, –NPK, 75N, and NPK. For all 14 of these QTLs, the WTR-1 alleles were significantly associated with key component traits in NuUE, but, in the case of chromosome 10, associated with two QTLs; these were contributed by the Hao-an-nong (HAN) allele in –N, –P, –NP and, –NPK conditions. These results indicated that the promising yield-related traits might share a similar genetic basis under different combinations of NPK treatments.

In previous studies, numerous mapping populations have been used to identify the QTLs associated with NPK deficiency tolerance traits in rice [26,28,29,31,34,48,52,55,58,61,67,74,75,76,111]. For each chromosome level, we identified seven or more QTLs located on chromosomes 2, 5, 8, and 10 and PVE ranged from 20.25% to 34.68%, and they were significantly associated with 1000-Gwt, PFP, FY, and PSPF under nutrient-deficient conditions of –P, –N, –NP, and –NPK. Several researchers in separate studies mentioned that chromosomes 2, 5, 8, and 10 were markedly associated with several agronomic traits, such as GY, nitrogen use efficiency (NUE), agronomic nitrogen use efficiency (agNUE), PSPF, nitrogen absorption ability (NAA), nitrogen content in shoots (NCS), harvest index (HI), root dry weight (RDW), shoot dry weight (SDW), and number of tillers (NT) QTLs under –N conditions [34,52,54,76]. Similarly, under low-P conditions, 36 QTLs were reported on the same chromosomes 2, 5, 8, and 10, respectively. These chromosomes were linked with phosphorus deficiency tolerance traits, such as phosphorus uptake (PUP), phosphorus use efficiency (PUE), phosphorus use efficiency for grain yield (PUEg), RDW, relative root length (RRL), panicle number per plant (PNPP), phosphors translocation (PT), phosphors translocation efficiency (PTE), spikelet fertility (SPF), 1000-Gwt, BY, relative root volume (RRV), and relative plant height (RPH), mapped in the different genetic backgrounds of mapping populations [48,55,56,59,60,61,67]. However, the major QTL, *Pup1,* was located on chromosome 12 at 54.5 cM, which explained PV of 78.8%, contributing to enhancing the uptake capacity of P from soils [31,55], and, further, the specified region of the 278-kbp sequence was significantly directly linked with P deficiency tolerance [112]. In this study, six QTLs were located on chromosome 12, with PVE ranging from 5.89% to 9.49%. Among these, one QTL for AE (*qAE_12.1*) was very close to this region (59.7cM), where *Pup1* is located on chromosome 12. Several researchers identified the same region as contributing toward tolerance of several biotic and abiotic stresses, such as drought, cold [97,113,114,115,116], and aluminum toxicity [116] in rice.

Interestingly, the identified associated QTLs governing seven traits (1000-Gwt, FGN, PSPF, BY, GY, AE, and PFP) on chromosome 6, under phosphorus-deficient conditions, significantly contributed to PVE ranging from 7.51% to 27.49%. Earlier independent studies of Ni et al. [31] and Wissuwa et al. [55] mapped major QTLs for RTA, RSDW, and RRDW on the same chromosome 6, and a group of P-responsive genes and transcription factors were also located in this region [113], which may confer tolerance of P deficiency [117]. However, in comparison to *Pup1* on chromosome 12, major QTLs and P-responsive genes were mostly located on chromosome 6, which indicates that both have independent genes and regulatory pathways [118].

In the deficiency of –N, –P, –NP, and –NPK conditions, four QTLs for GY were identified in –P (*qGY_2.4*, *qGY_4.2*, *qGY_8.4,* and *qGY_10.5*), two QTLs in –NP (*qGY_5.4* and *qGY_10.4*), and one QTL in –NPK (*qGY_5.3*), which were detected on different chromosomes (2, 4, 5, 8, and 10) from the analysis of 49 major QTLs. These QTLs were explained by their PV ranges from 20.25% to 25.28%. This was in contrast to Yue et al. [52], who reported three QTLs for 1000-Gwt on chromosome 3 and 7 and another three QTLs for GY located on chromosomes 1, 4, and 9, and the phenotypic variation explained by these QTLs ranged from 4.93% to 26.73% and 5.73% to 6.80%, respectively. However, in the present study, on chromosome 4, peak marker SNP_4_21833014 was found to be close to *qGYP-4* and was also flanked by RM273–RM241 [52]. In the present study of the genomic region of SNP markers from SNP_1_195334 to SNP_1_23839187 on chromosome 1, five QTLs were identified (*qBY*, *qGY*, *qFGN*, *qPSPF*, and *q1000Gwt*), which explained PV ranging from 7.18% to 26.83% under N-deficient conditions. However, in the same genomic region of chromosome 1, several QTLs governing relative grain yield, grain weight, 1000-Gwt, and nitrate transporter, such as *NRT 2.1* and *OsNRT2.3,b* are significantly involved in nitrogen deficiency tolerance and improving N uptake and enhancement of grain yield under nitrogen deficiency [119,120,121,122,123]

### 3.5. QTL Hotspots

Association with several traits within a single genomic region has immense potential value for the development of desired target trait enhancement through breeding and marker-assisted selection (MAS) applications. A single gene may affect more than two traits through pleiotropy or with closely linked genomic loci [124,125]. In the present study, 14 hotspot QTLs were identified to be located on chromosomes 1, 2, 3, 4, 5, 7, 8, 9, and 11, which had PVE ranges from 5.99% to 34.68% (Figure 3). Of these 14, we revealed the top four QTL harbor regions on chromosomes 3, 5, 9, and 11, and were designated as QTLs harbor-I to -IV. Each of these QTL harbor regions contained more than 10 QTLs on the same genomic regions of SNP markers, and significantly carried the positive allele from recipient parent WTR-1. The top four hotspot QTLs (a total of 45 QTLs) located on chromosomes 3, 5, 9, and 11 had PVE ranging from 6.09% to 26.97% and LOD values of 2.62 to 13.11. The four chromosomal (3, 5, 9, and 11) regions were shared by nitrogen (N) [34,52], phosphorus (P) [39,48,55,59,60,61], and potassium (K) [39] deficiency QTLs reported in different mapping populations in rice.

Significant reports exist on chromosomes 3, 6, and 11 involved in phosphorus deficiency tolerance in rice. Using BILs derived from inter-specific crosses of *Oryza sativa* L. X *O. rufipogon* Griff. [38], F_3_ lines from the crosses between P-deficiency-tolerant variety NERICA10 and sensitive variety Hitomebore [126], and 271 introgression lines [60], identified QTL clusters for SDW, BY, and P uptake on chromosomes 3 and 11, which showed high significance for P-deficiency tolerance. Comparison with previous studies showed a majority of the candidate genes for P-deficiency tolerance (*Pup1*) and *PSTOL1* located on chromosome 12 [63,112,118,127] and P-responsive genes (such as *OsPTF1*) on chromosome 6 [117]. Our present study revealed that a major region of chromosomes 3, 5, 9, and 11 might provide novel loci to discover candidate genes related to P-deficiency tolerance, and these genes/alleles might play a co-regulated role for P-deficiency tolerance. These significant results would be more helpful to breeders and biotechnologists to understand the genetic and molecular basis of the physiological mechanisms of P-deficiency tolerance in rice.

In addition, we compared the presently identified QTL harbor regions of chromosomes 3, 5, 9, and 11 with previously reported genes and loci by using Q-TARO (http://qtaro.abr.affrc.go.jp/), and those are significantly associated with soil stress tolerance mechanisms, such as uptake and transportation of various nutrient elements from the soil to roots and shoots [128,129,130,131,132,133,134,135]. In QTL harbor-I on chromosome 3 (32.86 Mb) and QTL harbor-II (21.5 Mb) on chromosome 5 regions, nine genes and four QTLs were identified, along with IDEF2, *OsHsfA4a*, and *OsZIP* [131,136,137], which are involved in Fe homeostasis, phosphate uptake and translocation, distribution of Zn content from roots to shoots, and tolerance of cadmium stress (Table S2). Four QTLs (*qRFWw3*, *n-p3*, *qDLR3*, and *qZNT-3*) associated with multiple traits of low nitrogen and zinc toxicity tolerance showed resistance to alkaline stress [26,98,106,138]. QTL harbor-III (13.78 Mb) and QTL harbor-IV (26.62 Mb) were located on chromosomes 9 and 11. Two QTLs (*qLBI-9* and *qALSRL-9*) and one gene (*OsSTR1*) located on chromosome 9 were associated with mycorrhizal formation and resistance to Fe and Al toxicity [105,133,139], whereas chromosome 11 contained two QTLs (*qDLR11* and *qRRE-11*) responsible for tolerance of Al toxicity and alkaline stress [106,140].

According to the physical positions of one of the QTL hotspot regions, SNP marker SNP_3_853802 was associated with the highest number of 14 QTLs that were located on chromosome 3, and were distributed as five QTLs in –P (*q1000gwt_3*, *qPFP_3*, *qBY_3*, *qFGN_3,* and *qGY_3*), two QTLs in –N (*q1000gwt_3* and *qGY_3*), two QTLs in –NP (*qBY_3* and *qGY_3*), three QTLs in -NPK (*q1000gwt_3*, *qBY_3,* and *qGY_3*), and one QTL in 75N and NPK (*q1000gwt_3*). Hạnh et al. [141] identified three hotspot regions with response to low-nitrogen QTLs, which were flanked by RM265-RM165 on chromosome 1, RM3199-RM514 on chromosome 3, and RM080-RM281 on chromosome 8, which were significantly associated with the traits as total fresh weight of leaf blades (FW), RDW, SDW, nitrogen concentration in sheaths plus stem (NS), nitrogen concentration in leaf blades (NL), plant height (PH), chlorophyll content index (CCI), NUE, physiological nitrogen use efficiency (pNUE), and agNUE in RIL populations of IR64 and Azucena. In another study, Senthilvel et al. [58], using doubled haploid (DH) lines of IR64/Azucena in a pot experiment with three N doses (native, 0 kg/ha^−1^; normal, 100 kg/ha^−1^; and high, 200 kg/ha^−1^), identified seven main-effect QTLs that were associated with NUE traits and plant grain yield on chromosome 3 [58]. In similar studies, with two different N fertilizer rates (0 N and 130–135 kg N ha^−1^), in field conditions with RILs derived from two *Oryza sativa*–ssp. *indica* rice varieties (Zhenshan97/Minghui63), Wei et al. [76] reported a major QTL for grain yield (*qRGY3*) on chromosome 3, and another two QTLs (*qRGY7* and *qRGY11*) on chromosomes 7 and 11, detected with PV of 10.8% in 2006 and 16.0% in 2007. Moreover, three QTLs (*qRBM9-1*, *qRBM9-2*, and *qRBM10*) for biomass yield on chromosomes 9 and 10 together explained 33.6% of the total phenotypic variation. However, in corroboration with Senthilvel et al. [58] and Wei et al. [76], QTL mapping studies on DH and RIL populations revealed that chromosome 3 holds a promising trait and was associated with nitrogen use efficiency (NUE) in rice. In P deficiency, several QTLs have been reported on chromosome 3 [55,60,61], on chromosome 5 [48,59,61], on chromosome 9 [59,60,61], and on chromosome 11 [48,60,61], which explained PV ranging from 4.4% to 16.6% in different mapping populations of BILs, ILs, RILs, and BC_2_F_3_ in rice. Under low K, Wu et al. [39] identified a total 21 QTLs, related to PH, TN, SDW, RDW, relative potassium concentration in plant (RKC), relative potassium use efficiency (RKUE), and relative potassium uptake (RKUP) on chromosomes 2, 3, 7, and 8, that collectively explained PV from about 8% to about 15% using DH populations. On chromosome 3, *qFGN_3.6* significantly associated with RTN and other traits, such as PH, SDW, RDW, and RKUP, were flanked by RG179-RG403 and RZ284-RZ394, contributing PV of 9.7% to 14.4% [39]. Thus, it is suggested that the identified QTL harbor regions support the reported NPK QTLs, and are refined with chromosomal regions of genes and QTLs that were involved in multiple stress tolerance and transportation of nutrient elements from soil to shoots and grain, which could be valuable information for the understanding of their genetic and physiological mechanisms in future molecular breeding programs in rice.

## 4. Materials and Methods

### 4.1. Plant Materials

**Figure 4:**
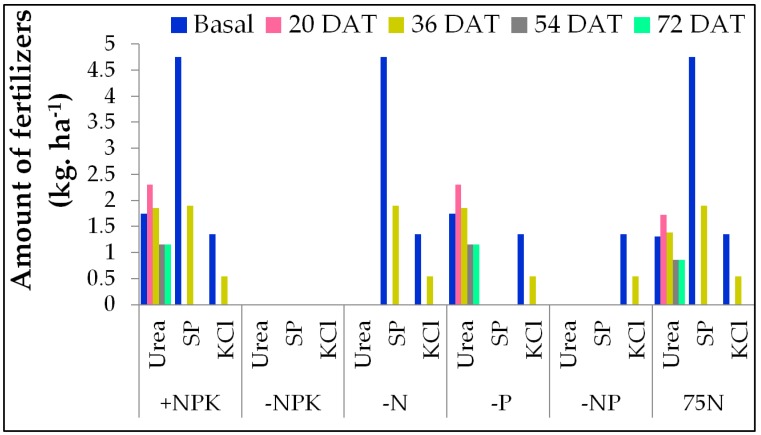
Application of fertilizers in six NuUE conditions with five splits.

A BC_1_F_5_ mapping population contained 230 introgression lines derived from a cross between Weed Tolerant Rice 1 (WTR-1), as the recipient parent, and Hao-an-nong (HAN), as the donor parent. The field experiments were carried out at the experimental farm of the International Rice Research Institute (IRRI), Los Baños, Laguna, Philippines (14.11° N, 121.15° E), during the 2014 dry season (DS). Seeds of 230 ILs, parents, and four checks (PSB Rc82, NSIC Rc222, Apo, and IR74371-70-1-1) were sown in a seedling nursery bed, and 21-day-old seedlings were transplanted with a single seedling per hill. The early generation of the backcross population (BC_1_F_2_) was grown in one generation under low-input, rainfed, and irrigation conditions during the 2011 wet season (WS), and was followed by four consecutive generations over the DS and WS during 2012 and 2013. Phenotypic screening for efficient selection was practiced based on higher grain yield under six nutrient conditions. Subsequently, this led to the development of 230 ILs. The detailed of the breeding strategies are described in Jewel et al [142]. The NuUE phenotyping experiment was laid out in an alpha lattice design with two replications, using a plot size of two rows × 12 plants/row, and a spacing distance of 0.2 × 0.2 m. NPK nutrients in the form of urea, superphosphate, and muriate of potash were applied at 160, 50, and 50 kg ha^−1^ in the DS, and at 90, 30, and 30 kg ha^−1^ in the WS, respectively. The checks and parents were replicated in all six nutrient conditions. The NPK fertilizers were applied five times in splits, as in basal level, and at 20, 36, 54, and 72 days after transplanting (DAT), respectively, against –N, –P, –NP, –NPK, 75N, and NPK conditions (Figure 4). Field management, including pest control, weeding, and irrigation, followed IRRI’s standard experimental farm practices to avoid adverse effects on grain quality.

### 4.2. Measurements of Agronomic and Yield-Attributed Traits

Each IL was selected in the middle row of plants, with three replications, and the plants were randomly sampled from each plot for phenotypic evaluation of six agronomic and yield-related traits. The six respective nutrient conditions, –N (PK fertilizer), –P (NK fertilizer), –NP (K fertilizer), –NPK (zero fertilizer), 75N (75% NPK fertilizer), and NPK (all nutrients), were recorded for seven prominent NuUE traits: agronomic efficiency (AE), partial factor productivity (PFP), grain yield (GY), biomass yield (BY), filled grains per plant (FGN), 1000-grain weight (1000-Gwt), and percentage of spikelet fertility (PSPF). Data analysis of the key phenotypic traits was performed with R software [143], and SPSS 17.0 software (IBM, Armonk, NY, USA) was used for the analysis of descriptive means, analysis of variance (ANOVA), Pearson correlation coefficient, and regression analysis, which were calculated among the ILs.

### 4.3. Calculation of Agronomic Efficiency (AE) and Partial Factor Productivity (PFP)

The increase in grain yield for each kg of fertilizer applied is known as AE. Using the formulas below, we calculated AE and PFP along with nitrogen fertilizer use condition. AE (kg kg^−1^) = grain yield (N fertilized–N unfertilized) in kg ha^−1^/Fertilizer N in kg ha^−1^ [144]. AE was computed for all the nutrient use efficient ILs, under six different nutrient conditions. The recommended fertilizer application rate was NPK = 160–50–50 kg ha^−1^. Among them, three possible AE formulas were depicted:

(i) AE _(N)_ = (Y_NPK_ − Y_0NPK_)/F_N_
(ii) AE _(N)_ = (Y_NK_ − Y_0NPK_)/F_N_
(iii) AE _(75N)_=(Y_(75N)_ − Y_0NPK_)/F_75N_

Similarly, PFP of applied nitrogen is also called nitrogen use efficiency. PFP (N) = YN (crop yield with applied N (kg ha^−1^)/amount of fertilizer N applied (kg ha^−1^) [144]. Based on applied NPK fertilizer, the performance of the selected and fixed nutrient use efficient ILs, across six different levels of nutrient conditions, was determined by the PFP of applied nitrogen using the following formula:

(iv) PFP_(N)_ = Y_(+NPK)_/F_N_
(v) PFP_(N)_ = Y_(−P)_/F_N_
(vi) PFP_(N)_ = Y(_75N)_/F_N_

Y_+NPK_ = crop yield with applied NPK fertilizer (kg ha^−1^); F_NPK_ = amount of fertilizer NPK applied (kg ha^−1^); F_N_ = fertilizer N in kg ha^−1^; AE = agronomic efficiency applied nitrogen; PFP = partial factor productivity applied nitrogen.

### 4.4. Genotyping via 6K SNP Array

A total of 230 ILs of plant genomic DNA were extracted from leaf tissue using the cetyl trimethylammonium bromide (CTAB) method [145] and quantified by a NanoDrop 8000 spectrophotometer (ThermoFisher Scientific, Waltham, MA, USA). The concentration of DNA was adjusted to 50 ng μL^−1^, and approximately 200 ng of DNA from each genotype used in the SNP array. The complete set of the BC_1_F_5_ mapping population, along with the parents used for the genotyping with 6K SNP array technology, was followed by DNA quantification, incubation, and hybridization of bead chips, staining, and image scanning according to the manufacturer’s instructions for the Illumina Infinium assay, and this work was conducted in the Genotyping Services Laboratory of the International Rice Research Institute. The resulting intensity data were processed using the genotyping module V2011.1 of Genome Studio software (Illumina Inc., San Diego, CA, USA) for SNP calling. The generated genotypic data indicated that 704 high-quality SNP markers were identified and further used in the construction of high-density linkage maps for NuUE QTLs, related to agronomic and yield-attributed traits.

### 4.5. Mapping of QTLs and Hotspot Regions for NuUE

The mapping of QTLs was carried out with IciMapping software (QTL IciMapping version 4.0) [146] using a single marker analysis (SMA) method. The permutation method was used to obtain an empirical threshold for claiming QTLs based on 1000 runs of randomly shuffling the trait values [147], and the logarithm of odds (LOD) value threshold for claiming QTLs was 2.5. The genetic distance (cM) between SNP marker positions was changed to physical distance (kb), with 1 cM equal to 260 kbp [148,149]. For seven promising critical agronomic and yield-related traits under six NuUE conditions, significant markers on the same chromosome, linkage disequilibrium (LD), and phenotypic trait data were calculated by the genetics package in R software [150]. A graphical representation of the linkage map was constructed using MapChart software [151]. Further, for fine-tuning of hotspot QTLs, we used the Q-TARO database (http://qtaro.abr.affrc.go.jp/) for the analysis of previously published co-localization of QTLs and genes related to soil stress tolerance in the rice genome.

## 5. Conclusions

In many plant breeding programs, the development of rice varieties with NuUE is being considered as a means to reduce the usage of NPK fertilizers by improving their use efficiency, thereby improving grain yield productivity. In the present study, selective introgression lines derived from Weed Tolerant Rice 1, as the recipient parent, and Hao-an-nong, as the donor parent, enabled us to identify a large number of QTLs (261 putative QTLs) linked with NuUE traits, of which 49 QTLs showed high PVE ranging from 20.25% to 34.68%. Among them, 22 QTLs reported as novel QTLs were responsible for PFP and AE traits. They were identified among the promising top four hotspot QTLs (QTLs harbor I-IV), which comprised more than ten QTLs associated with critical NuUE traits, such as 1000-Gwt, PFP, BY, FGN, GY, and PSPF. The hotspot regions of QTLs, expressed across all six NuUE conditions, suggested an underlying uniform basis of genetic mechanisms, contributing to the tolerance of these traits and associated with tightly linked genes or QTLs, or pleiotropic regulations. Corroboration with several research efforts, of the earlier discovered QTLs for NUE traits, revealed that most of the genomic region was confirmed and their positions precisely confirmed by significant SNP peak markers with high LOD and PVE values, and this could have potential value for introgression of the target using MAS. The list of QTLs and refined hotspot regions will facilitate further validation in systematic breeding for specific adaptability under low-input conditions, and suggest that hotspot genomic regions could be used as targets for a superior understanding of the NuUE mechanism and for improving NuUE traits in rice. In the future, identified promising harbor QTLs would be useful for developing elite introgression breeding lines comprising positive QTLs via marker-assisted selection, and also using them for carrying them forward by a pyramiding approach over the ILs, with desirable QTLs within the same population. Further, this will lead to fine-mapping and molecular cloning of the critical loci that will be useful for enhancing grain yield and quality under low-input fertilizer management conditions.

## Figures and Tables

**Table 1:** Statistical description of nutrient use efficiency-introgression lines for critical traits under six NPK combinations.

Traits	NuUE Condition	Mean ± Std. Error of Mean	Range (Min–Max)	SD	Variance (S^2^)	CV%
GY	NPK	32.48 ± 0.34	19.29–50.26	5.21	27.19	16.04
	75N	32.58 ± 0.35	20.82–48.83	5.44	29.69	16.70
	–N	21.61 ± 0.24	13.89–34.96	3.79	14.37	17.54
	–P	26.74 ± 0.31	17.57–42.19	4.78	22.86	17.88
	–NP	20.41 ± 0.29	11.86–40.43	4.49	20.20	22.00
	–NPK	20.49 ± 0.29	12.81–42.95	4.54	20.66	22.16
1000 Gwt	NPK	27.35 ± 0.13	19.20–31.75	2.05	4.21	7.50
	75N	27.35 ± 0.14	15.18–39.50	2.24	5.03	8.19
	–N	26.41 ± 0.11	20.65–30.60	1.82	3.31	6.89
	–P	27.13 ± 0.16	14.80–33.75	2.52	6.36	9.29
	–NP	26.88 ± 0.14	15.60–31.10	2.14	4.58	7.96
	–NPK	27.16 ± 0.12	21.50–33.35	1.83	3.37	6.76
PSPF	NPK	87.02 ± 0.28	68.05–95.24	4.34	18.84	4.99
	75N	87.14 ± 0.24	72.39–94.35	3.76	14.15	4.32
	–N	89.24 ± 0.29	68.50–96.59	4.44	19.78	4.98
	–P	87.60 ± 0.27	72.95–95.95	4.21	17.75	4.81
	–NP	89.93 ± 0.28	70.52–97.37	4.30	18.54	4.78
	–NPK	89.88 ± 0.26	77.27–97.75	4.09	16.76	4.55
FGN	NPK	1518.90 ± 17.95	956.83–2621.83	273.41	74,756.36	18.00
	75N	1402.42 ± 19.23	783.00–3177.50	293.04	85,876.01	20.90
	–N	1012.30 ± 11.88	686.50–1660.83	180.97	32,750.56	17.88
	–P	1318.12 ± 16.54	786.50–2251.17	252.01	63,512.83	19.12
	–NP	947.57 ± 13.26	445.33–1704.50	202.11	40,848.47	21.33
	–NPK	1040.31 ± 14.50	565.83–1675.83	220.94	48,814.58	21.24
BY	NPK	79.72 ± 1.14	35.92–137.23	17.43	303.83	21.86
	75N	210.24 ± 1.29	41.52–30,066.06	1968.81	3,876,231.42	936.46
	–N	50.47 ± 1.48	23.46–336.63	22.54	508.41	44.66
	–P	69.26 ± 1.18	38.31–144.27	18.07	326.83	26.09
	–NP	45.01 ± 0.84	18.16–89.43	12.90	166.49	28.66
	–NPK	47.29 ± 0.91	18.09–116.50	13.95	194.736	29.50

GY—grain yield; 1000-Gwt—1000-grain weight; PSPF—percentage of spikelet fertility; FGN—filled grains per plant; BY—biomass yield (BY); CV—coefficient of variance; SD—standard deviation.

**Table 2:** ANOVA for the testing of significance of genotype effect per fertilizer condition.

S. No.	Environment	Degrees of Freedom	Sum of Squares	Mean Squares	F-Value	Satterthwaite Denominator	Pr (>F)
1	NPK	230	12,493.16	54.56	1.07	34.38	0.4293
2	75N	230	12,736.71	55.62	1.20	451.78	0.0530
3	–N	230	6540.20	28.56	1.44	451.61	0.0005 ***
4	–P	230	9273.85	40.50	1.38	62.36	0.0658
5	–NP	230	9288.00	40.56	1.76	229.00	0.0000 ***
6	–NPK	230	9503.51	41.50	2.47	37.76	0.0007 ***

Significant codes: 0 ‘***’, 0.001 ‘**’, 0.01 ‘*’ 0.0.

**Table 3:** Testing for significance of fertilizer and its combined effect with genotype using −2 log-likelihood ratio test.

Effect	Model	AIC	BIC	Log-Likelihood	Chi Square	Degrees of Freedom	Pr (>Chisq)
Environment	1	17,071.23	18,457.21	−8301.62			
	2	17,058.00	18,449.91	−8294.00	15.2284	1	0.0001 ***
Genotype X Environment	3	17,056.00	18,441.98	−8294.00			
	4	17,058.00	18,449.91	−8294.00	0	1	0.9992

AIC—Akaike information criterion; BIC—Bayes information criterion; YLD—Yield; Deg—Degrees of freedom; Env—Environment; Rep—Replication; Blck—Block; Model 1: YLD~1 + Deg + (1|Env) + (1|Rep:Env) + (1|Rep:Blck:Env) + (1|Deg:Env). Model 2: YLD~1 + Deg + (1|Rep:Env) + (1|Rep:Blck:Env) + (1|Deg:Env). Model 3: YLD~1 + Deg + (1|Env) + (1|Rep:Env) + (1|Rep:Blck:Env) + (1|Deg:Env). Model 4: YLD~1 + Deg + (1|Env) + (1|Rep:Env) + (1|Rep:Blck:Env).

**Table 4:** Determination of NPK fertilizer efficiency in ILs under experiment on NuUE.

**Agronomic Efficiency (AE)**			
AE applied nitrogen	AE formula	>15 kg grain kg^−1^ nitrogen applied	>Parents
AE(N) = grain yield (N fertilized–0NPK unfertilized) in kg ha^−1^/Fertilizer N in kg ha^−1^	AE(_N)_ = (Y_NPK_ − Y_0NPK_) ÷ F_N_	117	74
	AE(N) = (Y_NK_ − Y_0NPK_) ÷ F_N_	33	28
	AE(N) = (Y_75N_ − Y_0NPK)_ ÷ F_75N_	161	86
**Partial Factor Productivity (PFP)**			
PFP Applied Nitrogen	PFP formula	>50 kg grain kg^−1^ nitrogen applied	>Parents
PFP(N) = grain yield N fertilized in kg ha^−1^/Fertilizer N in kg ha^−1^	PFP_(N)_ = Y_(+NPK_) ÷ F_N_	16	25
	PFP_(N)_ = Y_(−P_) ÷ F_N_	4	61
	PFP_(N)_ = Y(_75N_) ÷ F_N_	151	117

**Table 5:** Distribution of 704 polymorphic single nucleotide polymorphism (SNP) markers distributed in across the 12 chromosomes, with their average distance, genome size, coverage percentage, genetic distance, and physical distance per cM.

S. No.	Chr	Marker No.	Average Distance (Kb)	Genome Size (Kb)	Genome Size (Gramene)	Coverage Percentage	Genetic Distance (cM)	Physical Distance per (Kb)
1	Chr01	76	564.0	42,492.4	43,270.92	98.20	181.8	238.01
2	Chr02	45	797.4	35,401.9	35,937.25	98.51	157.9	227.59
3	Chr03	72	497.7	35,824.4	36,413.81	98.38	166.4	218.83
4	Chr04	84	405.0	33,864.4	35,502.69	95.39	129.6	273.94
5	Chr05	50	479.8	29,100.3	29,958.43	97.14	122.3	244.96
6	Chr06	73	422.7	30,809.5	31,248.78	98.59	124.4	251.20
7	Chr07	74	393.9	28,942.5	29,697.62	97.46	118.6	250.40
8	Chr08	43	654.5	27,809.9	28,443.02	97.77	121.1	234.87
9	Chr09	41	521.7	21,348.9	23,012.72	92.77	93.5	246.13
10	Chr10	43	464.0	19,635.6	23,207.28	84.61	83.8	276.94
11	Chr11	58	492.2	28,312.7	29,021.10	97.56	117.9	246.15
12	Chr12	45	603.7	27,023.4	27,531.85	98.15	109.5	251.43

Chr—chromosome; Kb—kilo base pairs; cM—CentiMorgan.

**Table 6:** Putative QTLs identified for AE and PFP for six different nutrient conditions using 230 nutrient use efficient ILs.

S. No.	NuUE Condition ^a^	Trait ^b^	QTLs ^c^	Chr	Position (bp) ^d^	Peak Marker ^e^	LOD Value	PVE% ^f^	Additive Effect
1	–P	AE	*qAE_2.1*	2	542,635	SNP_2_542635	2.77	6.43	3.16
2	–P	AE	*qAE_4.1*	4	21,815,986	SNP_4_21815986	4.01	9.17	2.13
3	–P	AE	*qAE_6.1*	6	9,977,282	SNP_6_9977282	4.52	10.27	2.28
4	75N	AE	*qAE_12.1*	12	14,936,674	SNP_12_14936674	2.55	5.92	−2.80
5	–P	PFP	*qPFP_1.1*	1	20,345,712	SNP_1_20345712	8.64	18.71	2.87
6	–P	PFP	*qPFP_2.2*	2	4,481,943	SNP_2_4481943	11.68	24.44	−3.11
7	–P	PFP	*qPFP_3.1*	3	853,802	SNP_3_853802	8.93	19.28	3.05
8	–P	PFP	*qPFP_4.1*	4	21,833,014	SNP_4_21833014	10.59	22.44	3.03
9	–P	PFP	*qPFP_5.1*	5	5,588,965	SNP_5_5588965	3.39	7.81	2.10
10	–P	PFP	*qPFP_6.1*	6	9,977,282	SNP_6_9977282	8.07	17.60	2.71
11	–P	PFP	*qPFP_7.1*	7	28,234,334	SNP_7_28234334	7.35	16.16	2.82
12	–P	PFP	*qPFP_8.2*	8	8,437,588	SNP_8_8437588	9.90	21.14	−2.89
13	–P	PFP	*qPFP_9.2*	9	12,154,616	SNP_9_12154616	8.73	18.89	3.15
14	–P	PFP	*qPFP_10.2*	10	6,149,421	SNP_10_6149421	12.15	25.28	−3.16
15	–P	PFP	*qPFP_11.1*	11	1,706,087	SNP_11_1706087	5.93	13.25	2.29
16	75N	PFP	*qPFP_1.2*	1	23,091,103	SNP_1_23091103	5.89	13.17	3.45
17	75N	PFP	*qPFP_2.1*	2	4,342,883	SNP_2_4342883	9.44	20.25	−3.99
18	75N	PFP	*qPFP_3.2*	3	3,542,519	SNP_3_3542519	7.32	16.09	4.16
19	75N	PFP	*qPFP_4.1*	4	21,833,014	SNP_4_21833014	7.60	16.66	3.68
20	75N	PFP	*qPFP_5.2*	5	15,469,279	SNP_5_15469279	9.78	20.91	−4.05
21	75N	PFP	*qPFP_6.2*	6	12,183,428	SNP_6_12183428	4.46	10.14	2.92
22	75N	PFP	*qPFP_7.2*	7	28,303,039	SNP_7_28303039	7.21	15.89	4.04
23	75N	PFP	*qPFP_8.1*	8	322,877	SNP_8_322877	7.09	15.64	−3.50
24	75N	PFP	*qPFP_9.1*	9	12,154,616	SNP_9_12154616	7.87	17.19	4.23
25	75N	PFP	*qPFP_10.1*	10	146,531	SNP_10_146531	9.13	19.68	−3.92
26	75N	PFP	qPFP_11.2	11	2,514,115	SNP_11_2514115	3.66	8.41	2.57

^a^ NPK condition trait: –P (negative phosphorus) and 75N (75% of nitrogen). ^b^ Trait name: AE (agronomic efficiency), PFP (partial factor productivity). ^c^ Name of identified QTL. ^d^ Nucleotide position (bp) of the SNP detected on each chromosome. ^e^ Peak marker of identified QTL. ^f^ Explanation of phenotypic variation.

## Supplementary Materials

Supplementary materials can be found at http://www.mdpi.com/1422-0067/20/4/900/s1.
