# Supplementary material for: Identification of Quantitative Trait Loci Associated with Nutrient Use Efficiency Traits, Using SNP Markers in an Early Backcross Population of Rice (Oryza sativa L.)
Source: Int J Mol Sci. 2019 Feb 19;20(4):900. doi: 10.3390/ijms20040900 (PMC6413108; doi:10.3390/ijms20040900)
Supplement: Supplementary file 1 [file ijms-20-00900-s001.pdf]

[IJMS] Manuscript ID: ijms-385561

Supplementary Materials: Tables accompanying the manuscript

Identification of quantitative trait loci associated with nutrient use efficiency traits using SNP markers in an early backcross population of rice (*Oryza sativa* L.)

Zilhas Ahmed Jewel [1, §], Jauhar Ali[1,*, §], Anumalla Mahender [1, §], Jose Hernandez[2], Yunlong Pang[1, 3], and Zhikang Li[4]

Supplementary Table 1 List of total putative QTLs for nutrient use efficient traits under six NPK combinations.

S. No	Traits	QTLs	NuUE condition	Chr	Position (bp)	SNP maker	LOD value	PVE%	Additive effect
1	1000-	*q1000Gwt_1.1*	-NP	1	932866	SNP_1_932866	3.46	7.96	-0.85
2	Gwt	*q1000Gwt_1.2*	-P	1	1982774	SNP_1_1982774	2.56	5.95	-0.81
3		*q1000Gwt_1.3*	75N	1	20706894	SNP_1_20706894	4.47	10.17	0.73
4		*q1000Gwt_1.4*	-NPK	1	20706894	SNP_1_20706894	13.53	27.71	1.01
5		*q1000Gwt_1.5*	NPK	1	20706894	SNP_1_20706894	3.07	7.11	0.59
6		*q1000Gwt_1.6*	-N	1	23839187	SNP_1_23839187	13.02	26.83	1.01
7	PFP	*qPFP_1.1*	-P	1	20345712	SNP_1_20345712	8.64	18.71	2.87
8		*qPFP_1.2*	75N	1	23091103	SNP_1_23091103	5.89	13.17	3.45
9	BY	*qBY_1.1*	-N	1	195334	SNP_1_195334	3.11	7.18	-3.78
10		*qBY_1.2*	-NPK	1	12216652	SNP_1_12216652	3.72	8.53	7.45
11		*qBY_1.3*	-NP	1	20238919	SNP_1_20238919	3.54	8.13	4.01
12		*qBY_1.4*	-P	1	20345712	SNP_1_20345712	4.01	9.17	5.98
13	FGN	*qFGN_1.1*	-N	1	195334	SNP_1_195334	4.50	10.23	-70.26
14		*qFGN_1.2*	-NPK	1	11576126	SNP_1_11576126	4.96	11.22	119.03
15		*qFGN_1.3*	-NP	1	11576126	SNP_1_11576126	4.67	10.59	106.77
16	GY	*qGY_1.1*	-NPK	1	12216652	SNP_1_12216652	3.44	7.92	2.42
17		*qGY_1.2*	-P	1	20345712	SNP_1_20345712	8.63	18.71	2.29
18		*qGY_1.3*	-N	1	20706894	SNP_1_20706894	3.11	7.18	1.10
19		*qGY_1.4*	75N	1	23091103	SNP_1_23091103	5.89	13.17	2.07
20	PSPF	*qPSPF_1.1*	75N	1	729666	SNP_1_729666	5.35	12.04	1.62
21		*qPSPF_1.2*	-N	1	20238919	SNP_1_20238919	8.16	17.78	2.03
22		*qPSPF_1.3*	-NPK	1	20238919	SNP_1_20238919	8.66	18.75	1.93
23		*qPSPF_1.4*	-NP	1	20238919	SNP_1_20238919	7.21	15.89	1.80
24		*qPSPF_1.5*	-P	1	20706894	SNP_1_20706894	4.19	9.55	1.40
25	1000-	*q1000Gwt_2.1*	75N	2	1660713	SNP_2_1660713	3.30	7.62	0.98
26	Gwt	*q1000Gwt_2.2*	NPK	2	1660713	SNP_2_1660713	4.77	10.82	1.13
27		*q1000Gwt_2.3*	-P	2	1660713	SNP_2_1660713	2.65	6.16	1.05
28		*q1000Gwt_2.4*	-NPK	2	4481943	SNP_2_4481943	17.76	34.68	-1.07
29		*q1000Gwt_2.5*	-NP	2	4481943	SNP_2_4481943	12.54	25.97	-1.17
30		*q1000Gwt_2.5*	-N	2	5830265	SNP_2_5830265	13.73	28.05	-1.10
31	AE	*qAE_2.1*	-P	2	542635	SNP_2_542635	2.77	6.43	3.16
32	PFP	*qPFP_2.1*	75N	2	4342883	SNP_2_4342883	9.44	20.25	-3.99
33		*qPFP_2.2*	-P	2	4481943	SNP_2_4481943	11.68	24.44	-3.11
34	BY	*qBY_2.1*	-NP	2	316859	SNP_2_316859	2.85	6.62	-6.19

35		*qBY_2.2*	-NPK	2	4342883	SNP_2_4342883	5.19	11.7	-4.54
36		*qBY_2.3*	-P	2	4481943	SNP_2_4481943	8.08	17.63	-7.85
37		*qBY_2.4*	-N	2	7076671	SNP_2_7076671	4.58	10.40	-3.59
38	FGN	*qFGN_2.1*	-NP	2	17414544	SNP_2_17414544	3.78	8.66	96.56
39	GY	*qGY_2.1*	-NP	2	3830219	SNP_2_3830219	8.32	18.08	-1.93
40		*qGY_2.2*	75N	2	4342883	SNP_2_4342883	9.44	20.25	-2.39
41		*qGY_2.3*	-NPK	2	4342883	SNP_2_4342883	8.55	18.54	-1.93
42		*qGY_2.4*	-P	2	4481943	SNP_2_4481943	11.68	24.44	-2.49
43	PSPF	*qPSPF_2.1*	-N	2	3830219	SNP_2_3830219	3.93	8.99	-1.33
44		*qPSPF_2.2*	-NPK	2	3830219	SNP_2_3830219	4.86	11.01	-1.36
45		*qPSPF_2.3*	-NP	2	3830219	SNP_2_3830219	3.35	7.72	-1.15
46		*qPSPF_2.4*	-P	2	20824958	SNP_2_20824958	6.04	13.49	2.53
47	1000-	*q1000Gwt_3.1*	75N	3	853802	SNP_3_853802	5.43	12.21	0.84
48	Gwt	*q1000Gwt_3.2*	-N	3	853802	SNP_3_853802	12.81	26.45	1.06
49		*q1000Gwt_3.3*	-NPK	3	853802	SNP_3_853802	10.47	22.21	0.95
50		*q1000Gwt_3.4*	NPK	3	853802	SNP_3_853802	3.11	7.19	0.62
51		*q1000Gwt_3.5*	-P	3	853802	SNP_3_853802	11.88	24.8	1.42
52		*q1000Gwt_3.6*	-NP	3	1670761	SNP_3_1670761	8.23	17.92	1.12
53	PFP	*qPFP_3.1*	-P	3	853802	SNP_3_853802	8.93	19.28	3.05
54		*qPFP_3.2*	75N	3	3542519	SNP_3_3542519	7.32	16.09	4.16
55	BY	*qBY_3.1*	-NPK	3	853802	SNP_3_853802	4.31	9.83	4.60
56		*qBY_3.2*	-NP	3	853802	SNP_3_853802	3.03	7.00	3.78
57		*qBY_3.3*	-P	3	853802	SNP_3_853802	4.17	9.51	6.37
58	FGN	*qFGN_3.1*	-P	3	853802	SNP_3_853802	3.33	7.67	75.96
59		*qFGN_3.2*	-NPK	3	1270943	SNP_3_1270943	3.48	8.01	116.99
60		*qFGN_3.3*	75N	3	16294363	SNP_3_16294363	5.00	11.3	93.25
61		*qFGN_3.4*	-N	3	16294363	SNP_3_16294363	3.85	8.82	51.86
62		*qFGN_3.5*	NPK	3	16996623	SNP_3_16996623	3.90	8.93	87.95
63		*qFGN_3.6*	-NP	3	33713486	SNP_3_33713486	4.16	9.49	84.63
64	GY	*qGY_3.1*	-N	3	853802	SNP_3_853802	3.46	7.97	1.22
65		*qGY_3.2*	-NPK	3	853802	SNP_3_853802	4.61	10.47	1.60
66		*qGY_3.3*	-NP	3	853802	SNP_3_853802	2.62	6.09	1.24
67		*qGY_3.4*	-P	3	853802	SNP_3_853802	8.93	19.28	2.44
68		*qGY_3.5*	75N	3	3542519	SNP_3_3542519	7.32	16.09	2.49
69	PSPF	*qPSPF_3.1*	-NP	3	1670761	SNP_3_1670761	6.87	15.2	1.87
70		*qPSPF_3.2*	-P	3	1670761	SNP_3_1670761	6.51	14.47	1.89
71		*qPSPF_3.3*	-N	3	3542519	SNP_3_3542519	11.97	24.96	2.58
72		*qPSPF_3.4*	-NPK	3	3542519	SNP_3_3542519	12.62	26.12	2.45
73		*qPSPF_3.5*	75N	3	16294363	SNP_3_16294363	4.79	10.84	1.28
74		*qPSPF_3.6*	NPK	3	16294363	SNP_3_16294363	5.67	12.71	1.51
75	1000-	*q1000Gwt_4.1*	-N	4	14609247	SNP_4_14609247	3.83	8.77	-0.58
76	Gwt	*q1000Gwt_4.2*	75N	4	17444232	SNP_4_17444232	3.54	8.13	-0.63
77		*q1000Gwt_4.3*	-NPK	4	17444232	SNP_4_17444232	3.68	8.45	-0.54
78		*q1000Gwt_4.4*	-NP	4	17444232	SNP_4_17444232	3.29	7.6	-0.64
79		*q1000Gwt_4.5*	NPK	4	21833014	SNP_4_21833014	3.85	8.82	0.64
80		*q1000Gwt_4.6*	-P	4	21833014	SNP_4_21833014	13.64	27.9	1.39
81	AE	*qAE_4.1*	-P	4	21815986	SNP_4_21815986	4.01	9.17	2.13
82	PFP	*qPFP_4.1*	75N	4	21833014	SNP_4_21833014	7.60	16.66	3.68
83		*qPFP_4.2*	-P	4	21833014	SNP_4_21833014	10.59	22.44	3.03

84	BY	*qBY_4.1*	-P	4	21833014	SNP_4_21833014	6.89	15.23	7.44
85	FGN	*qFGN_4.1*	NPK	4	14229856	SNP_4_14229856	3.32	7.65	77.43
86		*qFGN_4.2*	-NPK	4	31688380	SNP_4_31688380	3.10	7.16	-118.1
87	GY	*qGY_4.1*	75N	4	21833014	SNP_4_21833014	7.60	16.66	2.21
88		*qGY_4.2*	-P	4	21833014	SNP_4_21833014	10.59	22.43	2.43
89	PSPF	*qPSPF_4.1*	-N	4	3411505	SNP_4_3411505	4.28	9.75	-1.44
90		*qPSPF_4.2*	-NPK	4	3411505	SNP_4_3411505	3.28	7.57	-1.17
91		*qPSPF_4.3*	-NP	4	8085138	SNP_4_8085138	2.93	6.79	-1.16
92		*qPSPF_4.4*	-P	4	21833014	SNP_4_21833014	2.57	5.99	1.07
93	1000-	*q1000Gwt_5.1*	75N	5	5588965	SNP_5_5588965	4.78	10.83	0.85
94	Gwt	*q1000Gwt_5.2*	-N	5	5588965	SNP_5_5588965	13.11	26.97	1.16
95		*q1000Gwt_5.3*	-NPK	5	5588965	SNP_5_5588965	10.91	23.03	1.05
96		*q1000Gwt_5.4*	-NP	5	5588965	SNP_5_5588965	10.24	21.78	1.28
97		*q1000Gwt_5.5*	NPK	5	5588965	SNP_5_5588965	4.63	10.52	0.82
98		*q1000Gwt_5.6*	-P	5	5588965	SNP_5_5588965	10.85	22.92	1.48
99	PFP	*qPFP_5.1*	-P	5	5588965	SNP_5_5588965	3.39	7.81	2.10
100		*qPFP_5.2*	75N	5	15469279	SNP_5_15469279	9.78	20.91	-4.05
101	BY	*qBY_5.1*	-NPK	5	5789766	SNP_5_5789766	3.84	8.81	-7.12
102		*qBY_5.2*	-N	5	15469279	SNP_5_15469279	4.62	10.49	-3.60
103		*qBY_5.3*	-NP	5	15469279	SNP_5_15469279	7.19	15.83	-5.14
104		*qBY_5.4*	-P	5	15469279	SNP_5_15469279	4.78	10.82	-6.15
105	FGN	*qFGN_5.1*	-NPK	5	5789766	SNP_5_5789766	4.47	10.17	-127.97
106	GY	*qGY_5.1*	-P	5	5588965	SNP_5_5588965	3.39	7.81	1.68
107		*qGY_5.2*	75N	5	15469279	SNP_5_15469279	9.78	20.91	-2.43
108		*qGY_5.3*	-NPK	5	15469279	SNP_5_15469279	9.97	21.26	-2.07
109		*qGY_5.4*	-NP	5	15469279	SNP_5_15469279	9.92	21.18	-2.09
110	PSPF	*qPSPF_5.1*	-N	5	5588965	SNP_5_5588965	4.33	9.87	1.66
111		*qPSPF_5.2*	-NPK	5	5588965	SNP_5_5588965	3.57	8.19	1.40
112		*qPSPF_5.3*	-P	5	5588965	SNP_5_5588965	2.66	6.17	1.28
113		*qPSPF_5.4*	-NP	5	19516545	SNP_5_19516545	4.15	9.48	-1.28
114		*qPSPF_5.5*	75N	5	27086886	SNP_5_27086886	3.65	8.38	-2.31
115		*qPSPF_5.6*	NPK	5	27086886	SNP_5_27086886	3.03	7.02	-2.3
116	1000-	*q1000Gwt_6.1*	75N	6	13250266	SNP_6_13250266	5.41	12.17	0.78
117	Gwt	*q1000Gwt_6.2*	-N	6	13250266	SNP_6_13250266	10.26	21.81	0.9
118		*q1000Gwt_6.3*	NPK	6	13250266	SNP_6_13250266	9.29	19.98	0.97
119		*q1000Gwt_6.4*	-NPK	6	14441908	SNP_6_14441908	13.79	28.17	0.99
120		*q1000Gwt_6.5*	-NP	6	14441908	SNP_6_14441908	12.3	25.55	1.19
121		*q1000Gwt_6.6*	-P	6	14441908	SNP_6_14441908	13.4	27.49	1.39
122	AE	*qAE_6.1*	-P	6	9977282	SNP_6_9977282	4.52	10.27	2.28
123	PFP	*qPFP_6.1*	-P	6	9977282	SNP_6_9977282	8.07	17.6	2.71
124		*qPFP_6.2*	75N	6	12183428	SNP_6_12183428	4.46	10.14	2.92
125	BY	*qBY_6.1*	-P	6	13250266	SNP_6_13250266	5.87	13.13	6.99
126		*qBY_6.2*	-N	6	21415912	SNP_6_21415912	4.16	9.49	3.68
127		*qBY_6.3*	-NP	6	30809492	SNP_6_30809492	3.19	7.37	4.11
128	FGN	*qFGN_6.1*	-P	6	1928403	SNP_6_1928403	3.74	8.59	-85.77
129		*qFGN_6.2*	-NP	6	9836381	SNP_6_9836381	2.52	5.87	-64.26
130		*qFGN_6.3*	-NPK	6	29416997	SNP_6_29416997	3.37	7.77	73.55
131	GY	*qGY_6.1*	-P	6	9977282	SNP_6_9977282	8.07	17.6	2.17
132		*qGY_6.2*	75N	6	12183428	SNP_6_12183428	4.46	10.14	1.75

133		*qGY_6.3*	-N	6	29416997	SNP_6_29416997	3.94	9.02	1.39
134		*qGY_6.4*	-NPK	6	30809492	SNP_6_30809492	3.71	8.51	1.53
135		*qGY_6.5*	-NP	6	30809492	SNP_6_30809492	3.63	8.35	1.54
136	PSPF	*qPSPF_6.1*	75N	6	1768006	SNP_6_1768006	2.61	6.07	-1.14
137		*qPSPF_6.2*	NPK	6	9836381	SNP_6_9836381	3.08	7.12	-1.45
138		*qPSPF_6.3*	-P	6	9836381	SNP_6_9836381	3.07	7.10	-1.48
139		*qPSPF_6.4*	-N	6	29056693	SNP_6_29056693	14.71	29.73	2.96
140		*qPSPF_6.5*	-NPK	6	29056693	SNP_6_29056693	14.73	29.76	2.75
141		*qPSPF_6.6*	-NP	6	29056693	SNP_6_29056693	8.06	17.57	2.14
142	1000-	*q1000Gwt_7.1*	-P	7	27806332	SNP_7_27806332	4.28	9.75	0.88
143	Gwt	*q1000Gwt_7.2*	-N	7	28234334	SNP_7_28234334	6.93	15.32	0.82
144		*q1000Gwt_7.3*	-NPK	7	28234334	SNP_7_28234334	5.83	13.05	0.73
145		*q1000Gwt_7.4*	-NP	7	28234334	SNP_7_28234334	5.92	13.24	0.93
146	PFP	*qPFP_7.1*	-P	7	28234334	SNP_7_28234334	7.35	16.16	2.82
147		*qPFP_7.2*	75N	7	28303039	SNP_7_28303039	7.21	15.89	4.04
148	BY	*qBY_7.1*	-N	7	1558687	SNP_7_1558687	3.37	7.76	4.53
149		*qBY_7.2*	-P	7	17422851	SNP_7_17422851	2.57	5.99	6.38
150		*qBY_7.3*	-NP	7	28303039	SNP_7_28303039	3.28	7.57	4.08
151	FGN	*qFGN_7.1*	NPK	7	3853141	SNP_7_3853141	3.14	7.25	-139.93
152		*qFGN_7.2*	-NP	7	28234334	SNP_7_28234334	2.63	6.11	56.64
153	GY	*qGY_7.1*	-P	7	28234334	SNP_7_28234334	7.34	16.15	2.26
154		*qGY_7.2*	75N	7	28303039	SNP_7_28303039	7.21	15.88	2.43
155		*qGY_7.3*	-N	7	28303039	SNP_7_28303039	3.10	7.16	1.19
156		*qGY_7.4*	-NPK	7	28303039	SNP_7_28303039	3.15	7.29	1.39
157		*qGY_7.5*	-NP	7	28303039	SNP_7_28303039	3.18	7.34	1.41
158	PSPF	*qPSPF_7.1*	75N	7	4569035	SNP_7_4569035	2.57	5.98	-1.3
159		*qPSPF_7.2*	-N	7	5704192	SNP_7_5704192	2.96	6.86	-1.25
160		*qPSPF_7.3*	-NPK	7	28303039	SNP_7_28303039	3.80	8.71	1.38
161		*qPSPF_7.4*	-NP	7	28303039	SNP_7_28303039	3.40	7.82	1.33
162	1000-	*q1000Gwt_8.1*	NPK	8	2887584	SNP_8_2887584	4.63	10.51	-0.71
163	Gwt	*q1000Gwt_8.2*	75N	8	4101332	SNP_8_4101332	5.83	13.04	-0.79
164		*q1000Gwt_8.3*	-P	8	9019202	SNP_8_9019202	14.24	28.93	-1.39
165		*q1000Gwt_8.4*	-N	8	10073191	SNP_8_10073191	14.56	29.47	-1.02
166		*q1000Gwt_8.5*	-NPK	8	10073191	SNP_8_10073191	15.31	30.73	-1.01
167		*q1000Gwt_8.6*	-NP	8	10073191	SNP_8_10073191	11.74	24.54	-1.14
168	PFP	*qPFP_8.1*	75N	8	322877	SNP_8_322877	7.09	15.64	-3.50
169		*qPFP_8.2*	-P	8	8437588	SNP_8_8437588	9.90	21.14	-2.89
170	BY	*qBY_8.1*	-NPK	8	322877	SNP_8_322877	2.57	5.99	-3.24
171		*qBY_8.2*	-N	8	2887584	SNP_8_2887584	2.91	6.74	-3.01
172		*qBY_8.3*	-P	8	8437588	SNP_8_8437588	5.33	12.01	-6.48
173		*qBY_8.4*	-NP	8	8580913	SNP_8_8580913	3.82	8.76	-3.85
174	FGN	*qFGN_8.1*	-NP	8	23719048	SNP_8_23719048	3.46	7.96	64.61
175		*qFGN_8.2*	-P	8	23719048	SNP_8_23719048	4.72	10.7	90.74
176	GY	*qGY_8.1*	75N	8	322877	SNP_8_322877	7.09	15.64	-2.10
177		*qGY_8.2*	-NPK	8	322877	SNP_8_322877	6.64	14.73	-1.72
178		*qGY_8.3*	-NP	8	389278	SNP_8_389278	6.57	14.58	-1.75
179		*qGY_8.4*	-P	8	8437588	SNP_8_8437588	9.9	21.14	-2.31
180		*qGY_8.5*	-N	8	23719048	SNP_8_23719048	3.00	6.94	1.15
181	PSPF	*qPSPF_8.1*	-NP	8	4310448	SNP_8_4310448	6.39	14.22	-1.59

182		*qPSPF_8.2*	-N	8	23719048	SNP_8_23719048	9.49	20.35	2.23
183		*qPSPF_8.3*	-NPK	8	23719048	SNP_8_23719048	8.45	18.34	1.96
184		*qPSPF_8.4*	-P	8	23719048	SNP_8_23719048	7.48	16.43	1.95
185	1000-	*q1000Gwt_9.1*	75N	9	12154616	SNP_9_12154616	4.78	10.84	0.82
186	Gwt	*q1000Gwt_9.2*	-N	9	12154616	SNP_9_12154616	11.82	24.69	1.07
187		*q1000Gwt_9.3*	-NP	9	15446817	SNP_9_15446817	9.62	20.61	1.21
188		*q1000Gwt_9.4*	NPK	9	21215424	SNP_9_21215424	5.72	12.82	-0.75
189	PFP	*qPFP_9.1*	75N	9	12154616	SNP_9_12154616	7.87	17.19	4.23
190		*qPFP_9.2*	-P	9	12154616	SNP_9_12154616	8.73	18.89	3.15
191	BY	*qBY_9.1*	-NPK	9	7432853	SNP_9_7432853	3.22	7.44	6.96
192		*qBY_9.2*	-NP	9	12154616	SNP_9_12154616	2.96	6.86	3.91
193		*qBY_9.3*	-P	9	12154616	SNP_9_12154616	3.97	9.09	6.50
194	FGN	*qFGN_9.1*	-NPK	9	7432853	SNP_9_7432853	3.14	7.26	114.93
195		*qFGN_9.2*	-NP	9	7432853	SNP_9_7432853	4.22	9.62	122.15
196		*qFGN_9.3*	-P	9	12154616	SNP_9_12154616	3.65	8.39	82.93
197	GY	*qGY_9.1*	-NPK	9	7432853	SNP_9_7432853	2.60	6.04	2.12
198		*qGY_9.2*	75N	9	12154616	SNP_9_12154616	7.86	17.19	2.54
199		*qGY_9.3*	-NP	9	12154616	SNP_9_12154616	3.39	7.81	1.46
200		*qGY_9.4*	-P	9	12154616	SNP_9_12154616	8.73	18.89	2.52
201		*qGY_9.5*	-N	9	15322775	SNP_9_15322775	3.44	7.92	1.22
202	PSPF	*qPSPF_9.1*	-N	9	15446817	SNP_9_15446817	11.77	24.6	2.54
203		*qPSPF_9.2*	-NPK	9	15446817	SNP_9_15446817	11.78	24.61	2.36
204		*qPSPF_9.3*	-NP	9	15446817	SNP_9_15446817	6.85	15.14	1.88
205		*qPSPF_9.4*	-P	9	15446817	SNP_9_15446817	7.26	15.97	2.00
206	1000-	*q1000Gwt_10.1*	-NPK	10	6149421	SNP_10_6149421	16.28	32.32	-1.03
207	Gwt	*q1000Gwt_10.2*	-P	10	6149421	SNP_10_6149421	14.09	28.68	-1.38
208		*q1000Gwt_10.3*	-NP	10	8404652	SNP_10_8404652	13.56	27.77	-1.22
209		*q1000Gwt_10.4*	-N	10	9095431	SNP_10_9095431	14.5	29.37	-1.02
210		*q1000Gwt_10.5*	75N	10	11195773	SNP_10_11195773	4.14	9.46	-0.67
211		*q1000Gwt_10.6*	NPK	10	11195773	SNP_10_11195773	5.65	12.67	-0.75
212	PFP	*qPFP_10.1*	75N	10	146531	SNP_10_146531	9.13	19.68	-3.92
213		*qPFP_10.2*	-P	10	6149421	SNP_10_6149421	12.15	25.28	-3.16
214	BY	*qBY_10.1*	-NP	10	146531	SNP_10_146531	7.37	16.2	-5.20
215		*qBY_10.2*	-NPK	10	4281052	SNP_10_4281052	4.60	10.45	-4.29
216		*qBY_10.3*	-P	10	11664750	SNP_10_11664750	7.44	16.35	-7.56
217		*qBY_10.4*	-N	10	12145295	SNP_10_12145295	5.11	11.53	-3.81
218	FGN	*qFGN_10.1*	-NPK	10	18820606	SNP_10_18820606	4.52	10.28	-71.21
219		*qFGN_10.2*	-NP	10	18820606	SNP_10_18820606	4.13	9.42	-62.95
220	GY	*qGY_10.1*	75N	10	146531	SNP_10_146531	9.13	19.68	-2.35
221		*qGY_10.2*	-NPK	10	146531	SNP_10_146531	9.25	19.89	-2.00
222		*qGY_10.3*	-N	10	2056123	SNP_10_2056123	2.92	6.77	-1.01
223		*qGY_10.4*	-NP	10	5338202	SNP_10_5338202	9.55	20.46	-2.06
224		*qGY_10.5*	-P	10	6149421	SNP_10_6149421	12.15	25.28	-2.53
225	PSPF	*qPSPF_10.1*	-NPK	10	12048430	SNP_10_12048430	3.54	8.15	-1.18
226		*qPSPF_10.2*	-NP	10	12048430	SNP_10_12048430	3.30	7.61	-1.16
227		*qPSPF_10.3*	-N	10	18820606	SNP_10_18820606	5.38	12.1	-1.54
228		*qPSPF_10.4*	-P	10	18820606	SNP_10_18820606	3.29	7.59	-1.19
229	1000-	*q1000Gwt_11.1*	-N	11	1706087	SNP_11_1706087	6.07	13.55	0.69
230	Gwt	*q1000Gwt_11.2*	-NPK	11	1706087	SNP_11_1706087	7.29	16.04	0.73

231		*q1000Gwt_11.3*	-NP	11	1706087	SNP_11_1706087	5.49	12.33	0.81
232		*q1000Gwt_11.4*	-P	11	2514115	SNP_11_2514115	6.07	13.55	0.95
233		*q1000Gwt_11.5*	75N	11	22440795	SNP_11_22440795	3.97	9.09	-0.66
234		*q1000Gwt_11.6*	NPK	11	22440795	SNP_11_22440795	7.70	16.87	-0.87
235	PFP	*qPFP_11.1*	-P	11	1706087	SNP_11_1706087	5.93	13.25	2.29
236		*qPFP_11.2*	75N	11	2514115	SNP_11_2514115	3.66	8.41	2.57
237	BY	*qBY_11.1*	-NPK	11	1706087	SNP_11_1706087	3.19	7.36	3.60
238		*qBY_11.2*	-P	11	1706087	SNP_11_1706087	3.39	7.81	5.23
239		*qBY_11.3*	75N	11	22440795	SNP_11_22440795	3.19	7.37	-7.95
240		*qBY_11.4*	-N	11	22440795	SNP_11_22440795	3.88	8.89	-3.34
241		*qBY_11.5*	-NP	11	22440795	SNP_11_22440795	2.88	6.68	-3.36
242	FGN	*qFGN_11.1*	-NPK	11	2514115	SNP_11_2514115	4.61	10.47	71.93
243		*qFGN_11.2*	-NP	11	18483298	SNP_11_18483298	2.74	6.37	-68.5
244		*qFGN_11.3*	NPK	11	18483298	SNP_11_18483298	2.92	6.76	-93.15
245		*qFGN_11.4*	75N	11	28312708	SNP_11_28312708	5.00	11.30	93.25
246		*qFGN_11.6*	-P	11	28312708	SNP_11_28312708	7.19	15.84	99.49
247	GY	*qGY_11.1*	-NPK	11	1706087	SNP_11_1706087	5.25	11.82	1.54
248		*qGY_11.2*	-P	11	1706087	SNP_11_1706087	5.93	13.25	1.83
249		*qGY_11.3*	75N	11	2514115	SNP_11_2514115	3.66	8.41	1.54
250		*qGY_11.4*	-NP	11	2514115	SNP_11_2514115	4.07	9.29	1.39
251	PSPF	*qPSPF_11.1*	-NPK	11	1706087	SNP_11_1706087	8.64	18.72	1.77
252		*qPSPF_11.2*	-NP	11	1706087	SNP_11_1706087	5.14	11.61	1.41
253		*qPSPF_11.3*	-N	11	2514115	SNP_11_2514115	5.06	11.42	1.49
254		*qPSPF_11.4*	-P	11	25811598	SNP_11_25811598	2.65	6.16	-1.08
255		*qPSPF_11.5*	75N	11	28312708	SNP_11_28312708	4.79	10.84	1.28
256	1000-Gwt	*q1000Gwt_12.1*	NPK	12	2001943	SNP_12_2001943	4.16	9.49	0.87
257	AE	*qAE_12.1*	75N	12	14936674	SNP_12_14936674	2.55	5.92	-2.80
258	BY	*qBY_12.1*	-N	12	7445812	SNP_12_7445812	2.55	5.92	4.25
259		*qBY_12.2*	-NPK	12	7445812	SNP_12_7445812	3.19	7.37	5.65
260	FGN	*qFGN_12.1*	-NPK	12	3843753	SNP_12_3843753	3.07	7.09	83.67
261	GY	*qGY_12.1*	-NPK	12	5851455	SNP_12_5851455	2.53	5.89	1.54

Supplementary Table 2 Lists of available candidate genes and QTLs located in the QTL harbour-I to IV hotspot regions of four chromosomes (Q-TARO database)

S. No	Gene	Chr	Position (bp)		Function	Reference
			Genome start	Genome ending		
1	Pez1 (Phenolics efflux zero 1)	3	21553344	21560048	Localized in the plasma membrane and involved in Cadmium (Cd) and Iron (Fe) uptake in the stele.	[143]
2	OsIRO3	3	15551019	15553161	Fe homeostasis- upregulated under Fe deficiency in both root and shoot	[141]
3	Mit (Mitochondrial iron transporter)	3	10438693	10441586	Accumulation high levels of Fe in shoot under Fe deficiency	[152]
4	OsApx1 (Ascorbate peroxidase 1)	3	9893991	9897377	Strong effect on semi-dwarf phenotype and Aluminium tolerance	[153]
5	RPN10	3	7628772	7633071	Canavanine resistance-a subunit of 26S proteasome	[154]
6	OsFRDL1	3	6159182	6170117	Translocation of Fe	[139]
7	OsMTP8.1	3	6663914	6668886	Specific transporter for Mn tolerance in shoots.	[145]
8	OsGS1;2 (Glutamine synthetase 1;2)	3	6485246	6490063	Primary assimilation of ammonium ions taken up by rice roots	[146]
9	OsPT2 (Phosphate transporter2)	3	2793838	2795658	Phosphate uptake and translocation-involved significantly under Pi deprivation in roots and shoots.	[140]
10	IDEF2	5	20887132	20891253	Involved in iron homeostasis	[147]
11	OsZIP5	5	23262252	23264744	It's a zinc transporter played a role in Zn distribution fromroots to shoots	[142]
12	OsHsfA4a-TF	5	26405661	26408134	Associating with Cd tolerance by upregulation of metallothionein (MT) genes	[148]
13	STR1 (Stunted arbuscule 1)	9	14700465	14704135	ABC transporter for mycorrhizal formation	[144]

QTLs

S. No	QTLs	Chr	Position		Trait	Population	LOD value	PVE (%)	Reference
			Genome start	Genome ending					
1	qRFWw3	3	27894595	29301670	Root fresh weight (Well-watered) -PEG	RILs	8.12	16.88	[105]

					6000				
2	n-p3	3	23850041	25360678	Plant weight low N condition	RILs	15.23	5.95	[26]
3	qDLR3	3	13274763	21005826	Dead leaf rate at 55 days under alkaline stress	F_2	2.82	3.47	[113]
4	qZNT-3	3	696186	979543	Zn^{2+} toxicity tolerance	RILs	3.2	8.9	[149]
5	qLBI-9	9	18489616	21113859	Leaf bronzing index-Fe toxicity	CSSLs	2.72	11.16	[150]
6	qALSRL-9	9	20828464	21314308	Root length-tolerance to Al toxicity	DHs	6.28	18.3	[112]
7	qDLR11	11	383711	21835946	Dead leaf rate at 20 days under alkaline stress	F_2	2.78	5.96	[113]
8	qRRE-11	11	5402356	5699215	Relative root elongation-tolerance to Al toxicity	RILs	2.64	13.5	[151]

Supplementary Table 3 Lists of reported closely associated genes and QTLs for AE and PFP (QTARO databse)

S. No	Gene	Chr	Position (bp)		Functional roles	Reference
			Genome start	Genome ending		
1	BiP1 (Os06g0622700)	2	838730	842672	Seed storage protein and starch content and stress responses	[100]
2	BiP	2	838730	842672	Seed storage protein and starch content.	[99]
3	BiP3	2	838730	842672	BLB resistance.	[98]
4	OsHPL3	2	554579	556320	Resistance to rice brown planthopper, rice striped stem borer and Xanthomonasoryzaepv. oryzae."	[29]
5	OsHPR1	2	82952	80699	involved in photorespiratory metabolism	[101]
6	Pi2, Pi9	6	10386794	10389466	Resistance to blast disease	[103]
7	OsNOA1 (*Os02g0104700)*	2	261405	265160	NITRIC OXIDE-ASSOCIATED1- regulate chlorophyll biosynthesis, rubisco formation and plastid development	[102]
8	OsWRKY71	2	4542911	4544911	TFs, OsWRKY71, regulated by several defense signaling molecules, such as SA, methyl jasmonate and pathogen infection	[108]
9	Os4CL3	2	4280496	4285927	Abnormal anther development and disrupted lignin synthesis	[109]
10	OsGL1-2	2	4355042	4362385	Wax synthesis and stress resistance	[155]
11	OsWRKY45	5	14969392	14971539	BLB, Blast, sheath blight resistance drought, salinity and cold	[110,111,117]
12	OsAT1/Spl18	10	6320365	6325925	Resistance to blast disease	[116]

QTLs

S. No	QTLs	Chr	Position (bp)		Trait	Population	LOD value	PVE (%)	Reference
			Genome start	Genome ending					
1	qPHw6-2	6	6927624	20691040	Plant height (Well-watered)	RILs	7.23	11.43	[105]
2	qSFWd6	6	6927624	20691040	Shoot fresh	RILs	7.3	15.75	[105]

					weight (Water-deficit)				
3	amy6-1	6	4234080	28130383	Amylose content	RILs	21.8	39.6	[104]
4	qTN, qSDW, qRDW,	12	1548039	18160498	TN, SDW RDW, under low P	RILs	16.51, 16.98, 12.05	54.0, 60.8, 44.2	[31]
5	qDLA-12-3	12	2427030	24823647	Resistance to blast	DHs	5.34	17.5	[134]
6	qtl12.1	12	14257182	17758636	Biomass yield, PH	F3	23.0, 9.8	18.0, 5.0,	[106]
7	qtl12.2	12	9895474	17758636	Panicle number m-2, Flowering delay	F3	12.9, 19.0	9.01, 16.0	[106]
8	qtl12.1	12	14257182	17546401	Grain yield, Drought response index		34.0,39.0	33.0,37.0	[106]
9	qBT_4	4	19921036	29158662	Boron tolerance	RILs	10.92	-	[156]
10	qDSR 8	8	3927309	15529036	Dead seedling rate at 62 days under alkaline stress	F2	3.76	18.86	[113]
11	qALRR-8	8	5326640	17528755	Root length ratio under aluminium tolerance	DHs	8.23	28.7	[112]
12	qRRE-10	10	5167605	7599729	Relative root elongation under Al tolerance	BILs	3.33	11.8	[115]
13	qRFW	10	3758658	13115650	Relative fresh weight under ultraviolet-B resistance	BILs	15.5	40.8	[114]
14	qRCCL	10	3758658	13115650	Relative chlorophyll content of leaf blade under ultraviolet-B	BILs	13.9	36.7	[114]

References

152. Bashir, K.; Ishimaru, Y.; Shimo, H.; Nagasaka, S.; Fujimoto, M.; Takanashi, H.; Tsutsumi, N.; An, G.; Nakanishi, H.; Nishizawa, N.K. The rice mitochondrial iron transporter is essential for plant growth. *Nat Commun*. **2011**, 2, 322.
153. Rosa, S.B.; Caverzan, A.; Teixeira, F.K.; Lazzarotto, F.; Silveira, J.A.; Ferreira-Silva, S.L.; Abreu-Neto, J.; Margis, R.; Margis-Pinheiro, M. Cytosolic APx knockdown indicates an ambiguous redox responses in rice. *Phytochemistry*. **2010**, 71, 548-58.
154. Takase, T.; Yanagawa, Y.; Mitsuhara, I.; Ohashi, Y.; Hakagawa, H.; Hashimoto, J. Overexpression of a gene for 26S proteasome subunit RPN10 confers enhanced resistance to canavanine, an analog of arginine, in transgenic rice (*Oryza sativa* L.). *Plant Biotechnol.* **2004**, 21, 233–236.
155. Islam, M.A.; Du, H.; Ning, J.; Ye, H.; Xiong, L. Characterization of Glossy1-homologous genes in rice involved in leaf wax accumulation and drought resistance. *Plant Mol Biol.* **2009**, 70, 443-56.
156. Ochiai, K.; Uemura, S.; Shimizu, A.; Okumoto, Y.; Matoh, T. Boron toxicity in rice (*Oryza sativa* L.). I. Quantitative trait locus (QTL) analysis of tolerance to boron toxicity. Theor Appl Genet. **2008**, 117, 125-133.
